# Signatures of Topological States in Conjugated Macrocycles

**DOI:** 10.1021/acs.nanolett.3c04796

**Published:** 2024-04-09

**Authors:** Renad Almughathawi, Songjun Hou, Qingqing Wu, Colin J. Lambert

**Affiliations:** † Physics Department, Lancaster University, LA1 4YB Lancaster, United Kingdom; ‡ Physics Department, Faculty of science, Taibah University, Medina 42353, Saudi Arabia

**Keywords:** single-molecule junction, macrocycles, topological states

## Abstract

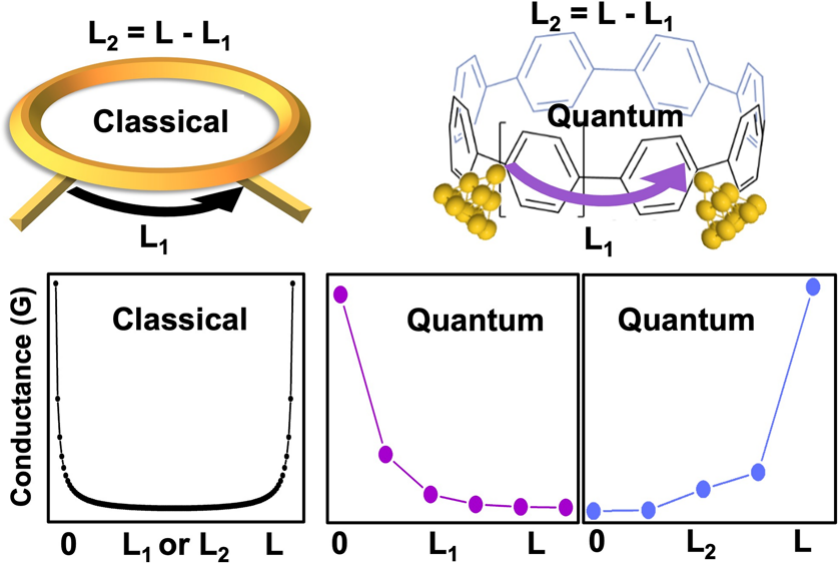

Single-molecule electrical junctions possess a molecular core connected to source and drain electrodes via anchor groups, which feed and extract electricity from specific atoms within the core. As the distance between electrodes increases, the electrical conductance typically decreases, which is a feature shared by classical Ohmic conductors. Here we analyze the electrical conductance of cycloparaphenylene (CPP) macrocycles and demonstrate that they can exhibit a highly nonclassical increase in their electrical conductance as the distance between electrodes increases. We demonstrate that this is due to the topological nature of the de Broglie wave created by electrons injected into the macrocycle from the source. Although such topological states do not exist in isolated macrocycles, they are created when the molecule is in contact with the source. They are predicted to be a generic feature of conjugated macrocycles and open a new avenue to implementing highly nonclassical transport behavior in molecular junctions.

During the past few years, studies of the flow of electricity through single molecules have revealed that intramolecular electron transport is controlled by quantum interference (QI), even at room temperature.^1^ As discussed in a variety of pedagogical accounts,^1−14^ QI occurs when multiple transport paths are present within a molecule that could be separated physically in real space or energetically separated in the Hilbert space of molecular orbitals. Constructive quantum interference (CQI) leads to high electrical conductance, whereas destructive quantum interference (DQI) leads to low electrical conductance and a variety of methods have been developed to control and switch between the these two states.^15−25^ Recently, the scale up of such QI effects to self-assembled molecular layers has even led to the realization of new thin-film materials whose cross-plane transport properties are controlled by QI.^26−29^ In contrast with the many studies of QI highlighted above, the role of topology in determining transport properties of single-molecule junctions has largely been ignored, despite the fact that in bulk 2D and 3D materials, new states of matter can emerge, such as topological insulators. The aim of the present paper is to highlight remarkable new quantum effects in molecular-scale devices, associated with topological properties of conjugated macrocycles, that lead to generic and highly nonclassical trends in the electrical conductance of macrocycles and related molecules. Conjugated macrocycles are cyclic chains of polyaromatic hydrocarbons, such as cycloparaphenylenes (CPPs), which consist of a number (*n*) of *para*-linked phenyl rings. CPPs have unique π-conjugated properties associated with their size,^30^ exhibiting a decrease in their HOMO–LUMO energy gap as the number of the phenyl ring decreases.^31,32^ Furthermore, CPPs can directly bind to a gold electrode via a (Au–C) covalent bond, which allows exploration of charge transport without introducing heteroatoms as anchors.^33,34^

In what follows, we highlight a remarkable nonclassical behavior in the length dependence of the electrical conductance of CPP molecules. To illustrate this behavior, consider first a classical ring of circumference *L* formed from two classical Ohmic conductors of length *L*_1_ and *L*_2_ = *L* – *L*_1_, shown in Figure 1a. If the electrical resistance per unit length of each branch is ρ, then the equivalent circuit is shown in Figure 1b. As shown in Figure 1d, the electrical conductance σ of such a ring is proportional to *L*/*L*_1_(*L* – *L*_1_), which is a minimum when *L*_2_ = *L*_1_ = *L*_2_ and diverges when a short circuit occurs at *L*_1_ = 0 or *L*_1_ = *L*. This classical result is in marked contrast with the electrical conductance of the macrocycle shown in Figure 1c, which increases or decreases monotonically with the length *L*_2_ or *L*_1_, as demonstrated in Figure 1e,g. The conductance shown in Figure 1e and 1g was obtained from a density functional theory (DFT)-based study (presented below) of electron transport through the macrocycle shown in Figure 1f, which contains 6 phenyl rings but possesses different connectivities to external electrodes. In what follows, we shall demonstrate that this remarkable nonclassical behavior is a generic feature of electron transport through CPP macrocycles and related molecules and is a signature of the topological nature of de Broglie waves produced by electrons injected into the CPP from external electrodes.

**Figure 1:**
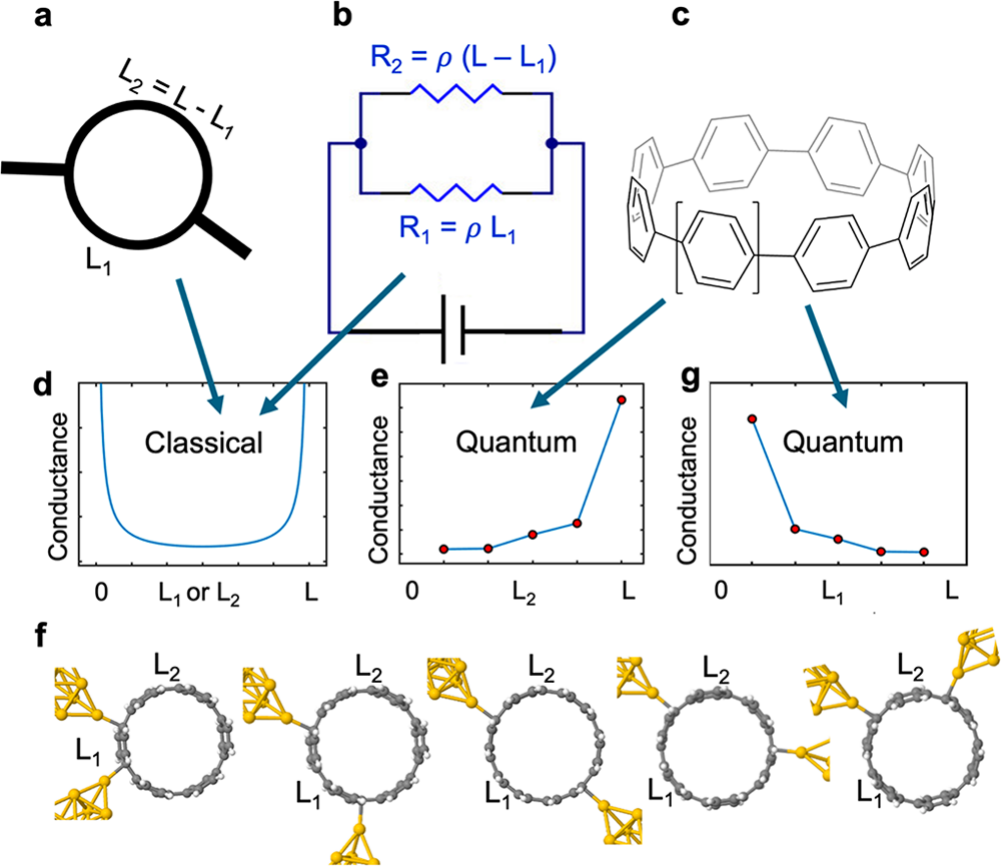
Schemes for contacting molecular junctions. (a) Classic ring with two branches. If the resistance per unit length of each branch is ρ, then the conductance of each branch is σ_1_ = 1/(ρ*L*_1_) and σ_2_ = 1/(ρ*L*_2_) and the conductance of the ring is . (b) An equivalent circuit for the classical ring of panel (a). (c) A macrocyle formed from phenyl rings connected by single bonds. (d) A plot of the electrical conductance of the classical ring of panel (a) versus the length *L*_1_ or *L*_2_ of one of the branches. (e) A plot of the electrical conductance of the series of macrocycles in panel (f) versus the length *L*_2_ of one of the branches, obtained using density functional theory. (f) A series of macrocycles, each formed from 6 phenyl rings (CPP6), but with different connectivities to external electrodes. (g) A plot of the electrical conductance of the series of macrocycles in panel (f) versus the length *L*_1_ of one of the branches, obtained using density functional theory. Note that there are many situations in single-molecule junctions where conductance decreases with increase of distance between electrodes. This is also a feature of classical (Ohmic) conductors. However, in contrast with this conventional behavior, the effect we describe is entirely the opposite, because as shown in panel (e), the conductance increases as the distance *L*_2_ between electrodes increases.

To explain the above behavior, we begin by analyzing a simple model that demonstrates the underlying mechanism. The above nonclassical conductance behavior in the macrocycle can be understood intuitively by examining the distribution of electrical currents (i.e., bond currents) in a tight-binding model (TBM, i.e., Hückel model) of the π system of CPPs, in which each π_*z*_ orbital is referred to as a “site”. To obtain a minimal model, which captures the underlying mechanism, each site is assigned a “site energy” ε_*c*_ and a nearest-neighbor interaction −γ, where γ > 0. Since ε_*c*_ can be regarded as defining the energy origin and γ can be regarded as defining the energy scale, the qualitative features of such a model are independent of the choice of these parameters. In what follows, we choose ε_*c*_ = 0 and γ = 1. Figure 2 shows the bond currents (obtained using the method outlined in section 8.10 of ref (13)) arising when electrons are injected by a source attached to the site indicated by a yellow star and removed by a drain attached to the site indicated by a pink star. To model a macrocycle, the structure in Figure 2 has periodic boundary conditions, such that the left-most site is connected to the right-most site by a bond −γ (not shown). As shown in Figure 2, the bond currents take the *L*_1_ = *L* – *L*_2_ path of the macrocycle, implying that path *L*_1_ behaves like a conducting wire, while the path *L*_2_ carries no current. As the insulating path *L*_2_ between the source (yellow star) and the drain (pink star) increases, the conducting path *L*_1_ = *L* – *L*_2_ becomes shorter, which results in a counterintuitive increase in electrical conductance. In what follows, we demonstrate that this interesting nonclassical behavior is a consequence of the topological nature of the electronic states (i.e., de Broglie waves) created by connecting source and drain to such macrocycles.

**Figure 2:**
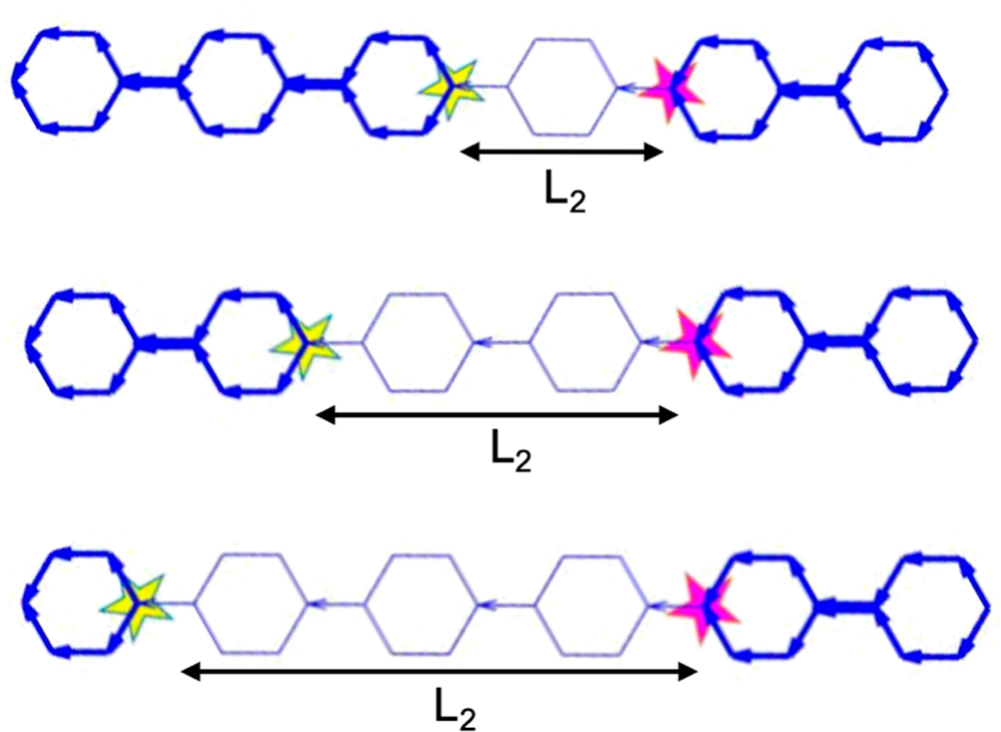
Bond currents of a six-ring macrocycle with a source marked by a yellow star and the drain marked by a pink star. The distance between the source and drain is denoted as *L*_2_, and the total length of the ring is *L*. *L*_1_ = *L* – *L*_2_ represents the length of the remaining part. To model a macrocycle, the structure has periodic boundary conditions, such that the left-most site of the first ring is connected to the right-most site of the last ring by a bond −γ (not shown).

Having demonstrated that the nonclassical behavior is captured by a simple TBM, we now show that the behavior is also predicted by DFT combined with quantum transport theory. To obtain the electrical conductance of a series of CPPs, the ground-state Hamiltonian of each optimized geometry was self-consistently obtained using the DFT-based package SIESTA.^35^ This was combined with the quantum transport code Gollum^36^ to obtain the electrical conductance of each system consisting of a left gold source electrode and a right gold drain electrode and the scattering region comprising the CPP. In what follows, the resulting DFT-based conductance trends are compared with the above TBM conductance trends. Figure 3a shows the numbering system used to label the sites of a tight-binding lattice of CPP6. The sites indicated by green and red circles are referred to as nodal sites. This is a bipartite lattice, where odd-numbered sites are connected to even-numbered sites only and vice versa, and furthermore, there are equal numbers of even and odd sites. To model a CPP ring, site 6 is connected to site 33. If site 6 is chosen as the electron injecting point, then when a drain is connected to a green nodal site (i.e., 12, 18, 24, 30, and 36), Figure 3e shows that DQI is obtained, indicated by a dip in the transmission coefficient at energy *E* = 0. In contrast, when a drain is connected to red nodal sites (i.e., 3, 9, 15, 21, 27, and 33), CQI is obtained, as indicated by the absence of a transmission dip near *E* = 0. This behavior is consistent with “magic number theory” (MNT),^4−6^ which also predicts DQI when the two electrodes are connected as even–even or odd–odd and CQI for even–odd connectivities to electrodes. MNT is described in detail in section 2.9 of ref (13) and outlined in the “Methods” (section S9 of the Supporting Information).

**Figure 3:**
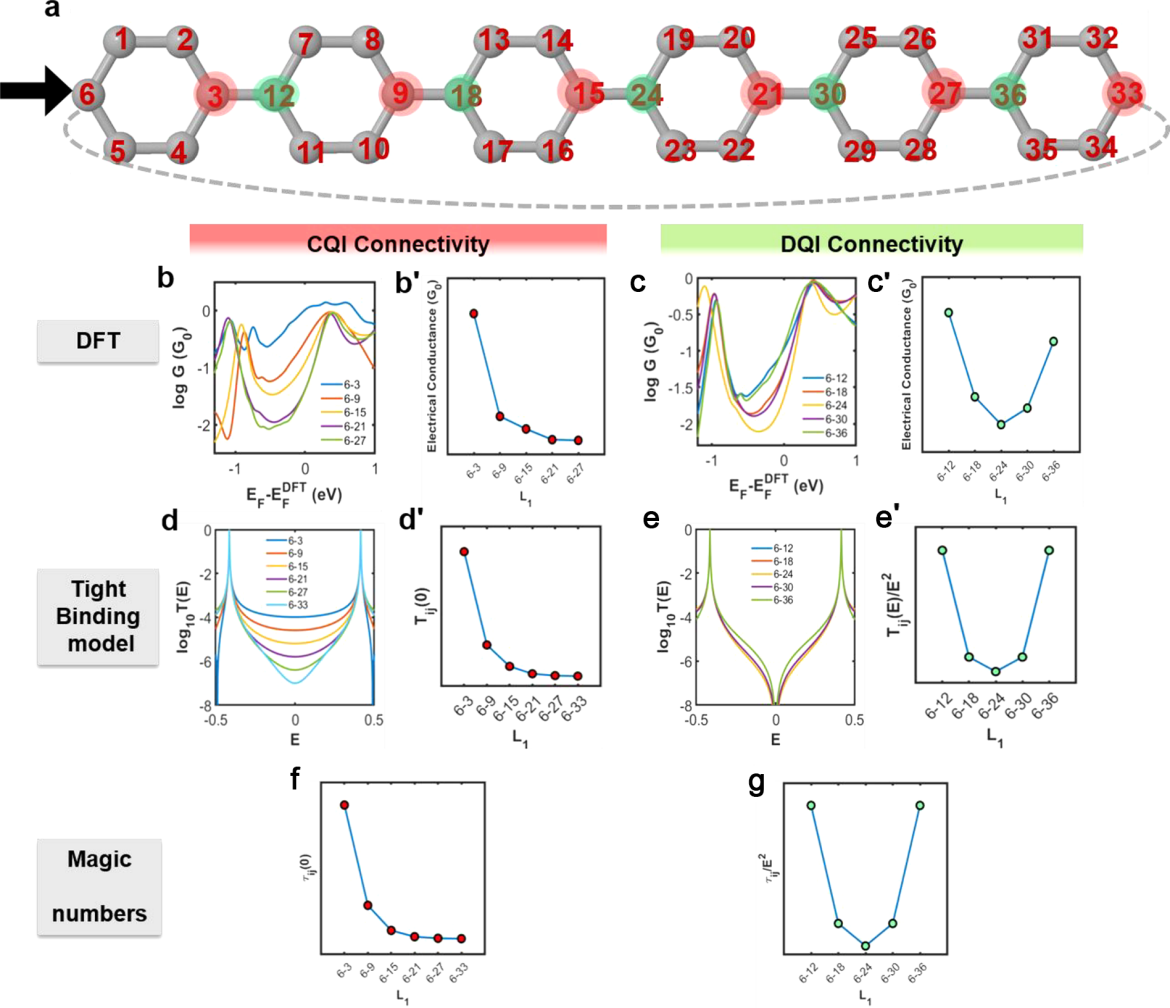
Transport properties for CPP6 in the level of DFT and TBM versus different connectivities (or separation *L*_1_ between each site in the connectivity pair) with the left electrode fixed to site 6. (a) A tight-binding lattice representing a 6-member macrocycle ring (CPP6). The Hamiltonian of this simple tight-binding model comprises single orbitals per atom with on-site energies ε_*c*_ = 0 and nearest-neighbor couplings = −1. This lattice is attached to two semi-infinite one-dimensional chains through weak couplings α = β = −0.1. (b, b′) DFT results of CQI electrical conductance versus the Fermi energy (*E*_*F*_) in unit of the quantum conductance *G*_0_ = 77 μS at room temperature 300 K and midgap conductance of panel (b) for different connectivity with length *L*_1_. It is of interest to plot *G* over a range of *E*_*F*_, to illustrate that the predicted nonclassical behavior is rather general and does not depend on the precise value of *E*_*F*_. (c, c′) DFT results of DQI electrical conductance as a function of the Fermi energy (*E*_*F*_) in units of the conductance quantum *G*_0_ = 77 μS at room temperature 300 K and midgap conductance of panel (c). (d, d′) TMB transmission curves *T*_*ij*_ (*E*) of CQI connectivity and midgap transmission coefficients *T*_*ij*_(0) of panel (d). (e, e′) TMB transmission curves *T*_*ij*_(*E*) of DQI connectivity transmission coefficient *T*_*ij*_(*E*)/*E*^2^ near *E* = 0 of panel (e). (f) MNT results of CQI connectivity τ_*ij*_(0) = [*M̅*_*ij*_]^2^ (see the magic number table for CQI connectivities of the macrocycle of Figure S4). (g) MNT results of DQI connectivity τ_*ij*_/*E*^2^ = [*D̅*_*ij*_]^2^ (see the magic number table for DQI connectivities of the macrocycle of Figure S5). The same connectivities for DFT and TBM are investigated except connectivity 6–33 in DFT calculation because the very close distance between the two electrodes will induce artifacts associated with direct electrode–electrode coupling.

For CQI connectivities, Figure 3b,d shows a comparison between the transport properties obtained using DFT and TBM. For the former case, the molecular junctions are built by forming a C–Au bond between a selected carbon atom of the CPP6 and the tips of gold source and drain electrodes.^15^ For the latter case, the transmission functions *T*_*ij*_(*E*) versus *E* are obtained by attaching 1D tight-binding leads to selected pairs of sites, labeled *i* and *j*. Clearly, the TBM results in Figure 3d reproduce the main features of DFT-based plots of Figure 3b, in which a decreasing trend is demonstrated as the drain electrode moves from site 3 to site 33, when the source electrode is fixed at site 6. In contrast, for the DQI connectivities present in Figure 3c (DFT results) and Figure 3e (TBM results), qualitatively different transport behavior is obtained, in which the conductance initially decreases when the drain moves from site 12 to site 24 and then increases as the drain moves to site 30 and further to site 36.

To further compare the DFT and TBM results, we plot the room-temperature electrical conductances versus separation *L*_1_ between the sites connected to the electrodes. Figure 3b′,d′,f shows that the DFT-based trends are in excellent agreement with predictions from both TBM and MNT. More details of the analysis of CPP2 and CPP6 are presented in sections S1–S3 of the Supporting Information. These results show that CQI connectivities lead to the nonclassical behavior shown in Figure 1e,g. Moreover, the decrease in conductance with increasing length persists even if the number of phenyl rings increases to CPP11, as shown in section S4 of the Supporting Information. In contrast, for the DQI case, the DFT results of Figure 3c′ resemble the classical behavior of Figure 1d, although unlike Figure 1d and the TBM and MNT results of Figure 3e′,g, the curve is not symmetric. In the case of DQI, since the TBM transmission coefficient *T*(*E*) is proportional to *E*^2^ near *E* = 0, the ratio *T*(*E*)/*E*^2^ remains finite at *E* = 0; therefore, to obtain qualitative trends, this is the quantity plotted in Figure 3e′,g.

To more completely characterize this nonclassical behavior, further calculations were carried out, in which the source is connected to site 5 of CPP6, as shown in Figure 3a and the drain is connected to different nodal sites (e.g., sites 12, 18, 24, 30, and 36 for CQI as shown in Figure 4 and sites 3, 9, 15, 21, 27, and 33 for DQI as shown in Figure S6). In this case, DFT, TBM and MNT results all show that the electrical conductance increases with increasing length *L*_1_, which is clearly nonclassical (Figure 4a–c).

**Figure 4:**
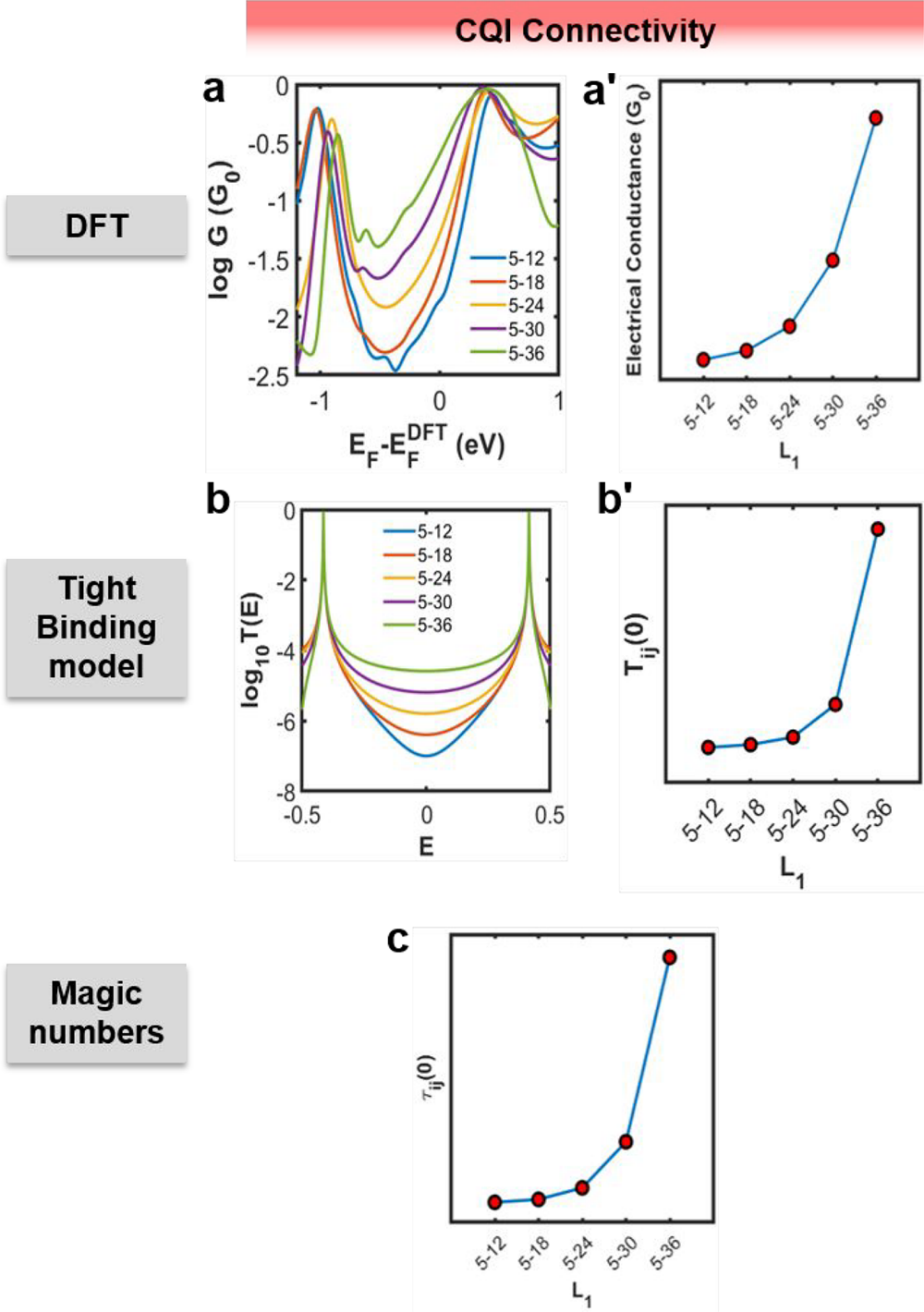
Transport properties for CPP6 in the level of DFT and TBM versus the connectivity (or the separation *L*_1_ between sites *i* and *j* along CPP6) with the left electrode fixed to site 5. (a, a′) CQI electrical conductance as a function of the Fermi energy (*E*_*F*_) in units of the quantum conductance *G*_0_ = 77 μS at room temperature 300 K and the midgap conductance of panel (a). (b, b′) CQI transmission curves *T*_*ij*_(*E*) for a tight-binding model of CPP6 of Figure 3a and the midgap transmission coefficients *T*_*ij*_(0) of panel (b) herein. (c) MNT-based results τ_*ij*_(0) = [*M̅*_*ij*_]^2^ versus the length *L*_1_ obtained from the magic number table of the macrocycle of Figure S4.

Unlike artificial quantum dots, whose connectivity to electrodes is not well-defined at an atomic scale, the connections of source and drain electrodes to a molecule in a single-molecule junction can be chosen to atomic accuracy. This is achieved by chemically attaching anchor groups to specific atoms of the molecule, which preferentially bind to the electrodes. For example, one such anchor group is the nitrogen atom of a pyridyl ring. In order to test the above nonclassical behavior experimentally, it will be necessary to incorporate anchor groups at specific points in the macrocycle. Therefore, to demonstrate that the above behavior is resilient and persists in the presence of anchor groups, we designed the series of molecules containing two nitrogen (N) heteroatoms, to which the gold electrodes would bind, as shown in Figure 5a. Following the numbering convention of Figure 3a, to obtain a series of molecules labeled *C*_*n*_ (*n* = 1, 2, 3, ... 5) with CQI connectivities, one N atom is placed on site 5 and a second N atom is placed on sites 8, 14, 20, 26, and 32 respectively. Similarly, Figure 5d shows a series of DQI connectivities labeled as *C*_*n*_ (*n* = 6, 7, ... 10). For these molecules, DFT and TBM results are similar to those of Figure 4, which shows that the nonclassical behavior of CPP macrocycles persists in the presence of pyridyl anchor groups.

**Figure 5:**
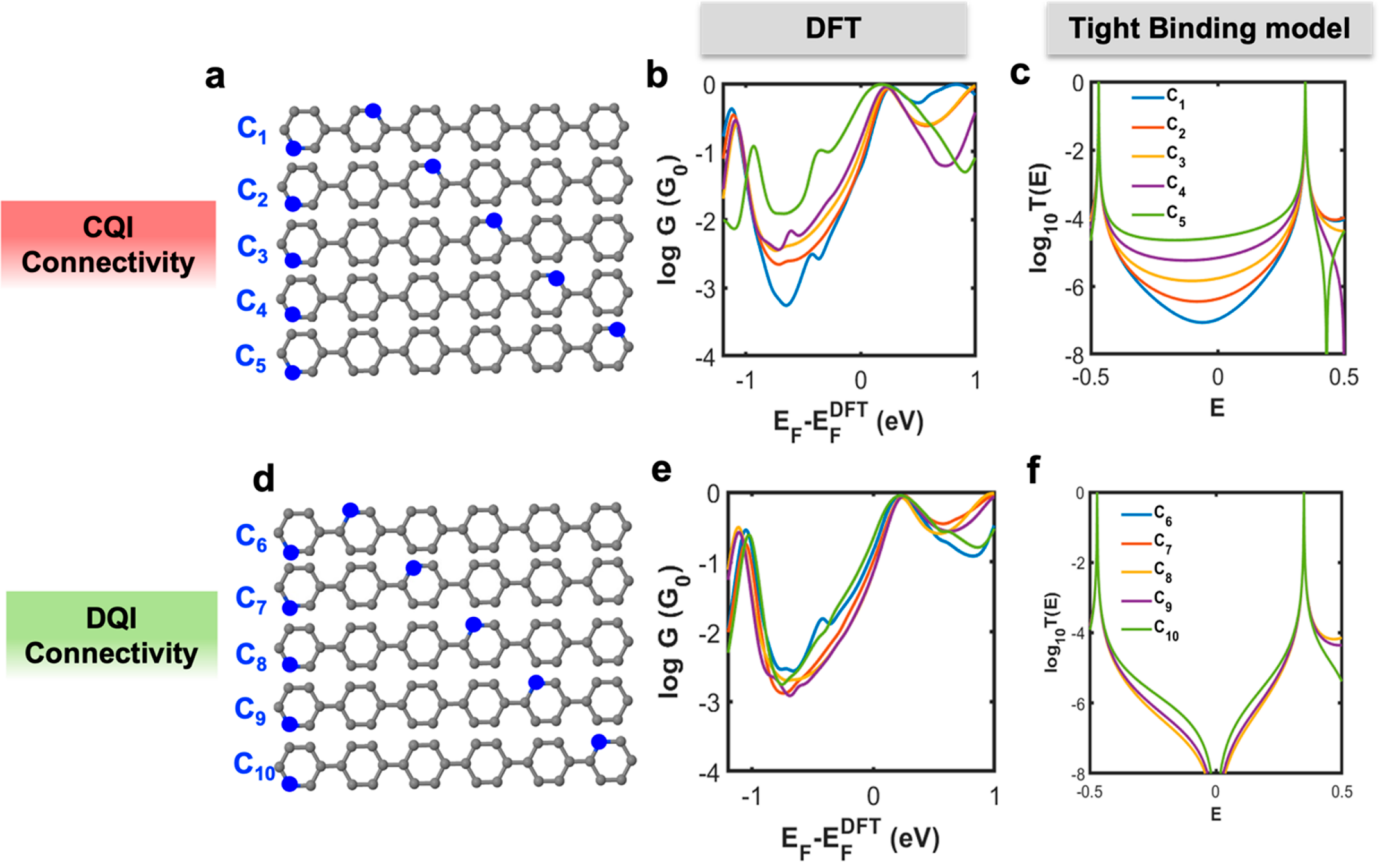
Transport properties for CPP6 in the level of DFT and TBM. (a) Schematic of CPP6 structure with CQI connectivity, where gray is assigned for carbon and blue for nitrogen. (b) CQI electrical conductance as a function of the Fermi energy (*E*_*F*_) in units of the quantum conductance *G*_0_ = 77 μS at room temperature 300 K. (c) CQI transmission curves *T*_*ij*_(*E*) for a tight-binding model of CPP6 in panel (a). (d) Schematic of CPP6 structure with DQI connectivity. (e) DQI electrical conductance as a function of the Fermi energy (*E*_*F*_) in units of the quantum conductance *G*_0_ = 77 μS at room temperature 300 K. (e) DQI transmission curves *T*_*ij*_(*E*) for a tight-binding model of CPP6 of panel (d). In TBM, the on-site energy of nitrogen, ε_n_, is −0.5, whereas that of carbon, ε_c_, is 0.

To highlight the topological origin of the above nonclassical behavior, it is useful to map the above-mentioned TBM of a macrocycle onto a linear chain of alternating bonds, by decimating the non-nodal sites, as described in section 12.2 of ref (13). For two phenyl rings, this is illustrated in Figure S14a and leads to an equivalent linear chain of alternating bonds, denoted as −α and −γ. At the center of the TBM HOMO–LUMO gap (i.e., *E* = 0), such decimation leads to α/γ = −2. For a linear chain of phenyl rings, one obtains the linear chain of alternating bonds shown in Figure S14b, with free ends, whereas for a macrocycle, one obtains the chain of alternating bonds shown in Figure S14c, with periodic boundary conditions. From a mathematical viewpoint, one could also consider the linear chain shown in Figure S14d, which is terminated by different bonds at each end. The lattices in Figure S14b–d are described by a Su, Schrieffer, and Heeger (SSH) model,^37^ with energy-dependent parameters −α and −γ. The SSH model is an example of a 1D topological insulator and the resulting edge states are a consequence of topological invariants associated with the underlying Hamiltonian.^38^ For *N* → ∞, as the ratio  is varied, the chain of Figure S14b exhibits a topological transition when  decreases below unity, at which point two exponentially decaying edge states appear at each end of the chain (i.e., the edge states appear at *E* = 0 when the chain is terminated by the weaker of the two bonds). For the chain of Figure S14d, this means that there will be an edge state on the left when  and an edge state on the right when . In our case, after decimation, , which means that the chain of Figure S14b contains no edge states, while that of Figure S14d contains an edge state on the right (more details are presented in section S7 of the Supporting Information).

Whatever the value of α obtained after decimation, Figure S14b shows that a topological edge state appears when an electrode is attached to a CPP macrocycle, even though the macrocycle itself is periodic and has no edges. More TBMs for the topological edge states of diatomic chains and rings are discussed in section S8 of the Supporting Information. This means that at the center of the HOMO–LUMO gap, the source electrode creates a state that decays exponentially either to the right or left of the atom attached to the source electrode, and it is this feature which leads to the nonclassical behavior of the transmission coefficient and electrical conductance (shown in Figures 1, 3, and 4) and to the nonclassical behavior of the currents, shown in Figure 2. This decaying state is clearly predicted by the magic number tables of CPP macrocycles, as shown, for example, by the exponentially decaying magic numbers highlighted in Figure S4. DFT calculations show that this behavior is robust and persists when nitrogen anchor groups are incorporated into the macrocycle.

In summary, we have demonstrated that the electrical conductance of a conjugated CPP macrocycle monotonically increases or decreases as the separation between two electrodes increases, which is highly nonclassical and in marked contrast with the behavior of an Ohmic ring of resistive material. To elucidate the origin of this effect from a different viewpoint, Figure 2 shows that the nonclassical effect is present in bond currents, whereas Figures 3 and 4 show that the nonclassical effect is also predicted by tight-binding theory, magic number theory, and density functional theory. Quantum interference effects are more ubiquitous than topological effects and therefore by studying quantum interference in macrocycles, it would be possible to discover this nonclassical conductance behavior without considering topological features. Indeed, we first noticed the nonclassical effect in the magic number tables associated with macrocycles and then proceeded to analyze its origin in more detail. For example, one could ask the following: In Figure 1e,g, why does the conductance increase with increasing *L*_2_ and decrease with increasing *L*_1_ and not the other way around? Similarly one could also ask why do the magic numbers highlighted in Figure S4 decrease from left to right and not vice versa? Our analysis shows that this nontrivial quantum behavior occurs because, when the CPP molecule is connected to a source electrode, a topological edge state is induced by the contact with a source, even though topological edge states do not exist in the isolated CPP.

It is interesting to note that topological effects in linear chains are discussed in refs (39−41), where it is demonstrated that the conductance of linear chains can increase with increasing length, because as the coupling between edge states at opposite ends of a linear molecule decreases, the HOMO–LUMO gap decreases. This increase in conductance of linear chains with length also occurs in porphyrin tapes,^42^ where it is also is associated with a decrease in the HOMO–LUMO gap with increasing length. Topological effects in 1D molecular wires, with diradical 2-butene and the diradical *p*-xylene building blocks, are discussed in ref (43), although their analysis of macrocycles is restricted to symmetric molecules in which the electrodes couple to diametrically opposite sites only. The present study is the first time that signatures of topology have been identified in the highly nonclassical electrical conductance behavior that arises when contacting a macrocycle of fixed length at different points along its circumference and opens the way for highly conductive single-molecule device designs, which utilize both quantum interference and topology.

## Supporting Information Available

Details of the calculations, DFT frontier orbital analysis and tight-binding model, additional transmission coefficient calculations (PDF)
