# Supplementary material for: Signatures of Topological States in Conjugated Macrocycles
Source: Nano Lett. 2024 Apr 9;24(16):4972–8. doi: 10.1021/acs.nanolett.3c04796 (PMC11057032; doi:10.1021/acs.nanolett.3c04796)
Supplement: Supplementary file 1 — nl3c04796_si_001.pdf [file nl3c04796_si_001.pdf]

Supporting Information

Signatures of topological states in conjugated macrocycles

*Renad Almughathawi[1,2], Songjun Hou[1], Qingqing Wu[1] * and Colin J. Lambert[1] **

1. Physics Department, Lancaster University, LA1 4YB Lancaster, United Kingdom.

2. Physics Department, Faculty of science, Taibah University, Medina, Saudi Arabia.

Contents

S1. Magic number theory and tight-binding transmission calculations of CPP2: application of the magic ratio rule (MRR). 2

S2. Frontier orbital analysis 3

S3. Magic-number theory and tight-binding transmission calculations of CPP6: application of the magic ratio rule (MRR). 4

S4. Transport properties of CPP11 obtained using both DFT and a tight-binding model: 7

S5. Magic number theory of a linear chain of phenyl rings....10

S6. Theory of edge states in macrocycles. 11

S7. The topological origin of the non-classical conductance behaviour of CPP macrocycles. 19

S8. Edge states of diatomic chains and rings based on TBMs. 22

S9. METHODS....23

Appendix A. Green's function due to a source at site lq. 27

Appendix B. Expressions for the full Green's function G. 28

Appendix C. Topological insulators. 33

Appendix D. Chiral symmetry. 34

Appendix E. Edge states and Green's functions. 35

Appendix F. Green's functions at small, but finite energy E. 37

Appendix G. Expressions for $m2$ for the chain of Figure S13. 38

Appendix H: Connectivity theory. 41

References 46

S1. Magic number theory and tight-binding transmission calculations of CPP2: application of the magic ratio rule (MRR).

As an example, consider the following tight-binding model of CPP2, whose Hamiltonian is proportional to a connectivity matrix C, with matrix elements C_{ij} for i odd and j even or vice versa and zero otherwise[1]. The connectivity table for CPP2 is shown in Figure S1b and the corresponding magic number table M, with matrix elements M_{ij} is given by $M = dC^{-1}$, where d is any convenient number. In Figure S1c, we have chosen $d = -3$, to obtain a magic number table containing integers only.

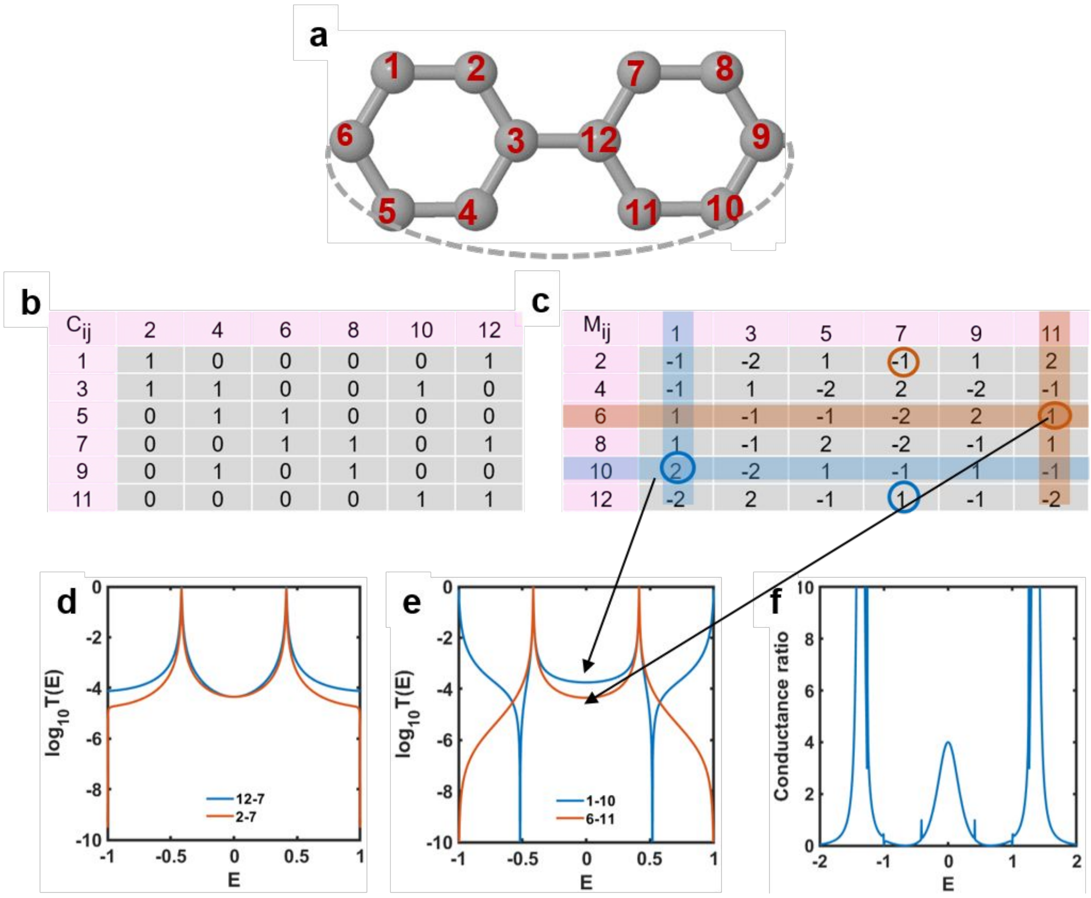

Figure S1. (a) A tight binding lattice representing a 2-ring macrocycle, showing the numbering system. (b,c) The connectivity and the magic number tables for the 6-ring macrocycle of figure S1a respectively. (d,e) Transmission curves $T_{ij}(E)$ for a tight-binding model of the 2-ring macrocycle of figure S1a. (f) The ratio from transmission curves of figure S1e which is satisfied the magic number ratio from the magic table of figure S1c.

According to magic number theory (MNT), the ratio of two electrical conductances G_{ij} and G_{lm} is predicted by a magic ratio rule (MRR) to be $\frac{G_{ij}}{G_{lm}} = (M_{ij}/M_{lm})^2$. In the case of CPP2,

from the magic number table in figure S1c, one obtains, for example.

$$\frac{G_{12,7}}{G_{2,7}} = \frac{(1)^2}{(-1)^2} = 1 \quad \text{and} \qquad \frac{G_{1,10}}{G_{6,11}} = \frac{(2)^2}{(1)^2} = 4$$

This is ratio is identical to the ratio of the two transmission functions shown in figure S1e.

S2. Frontier orbital analysis

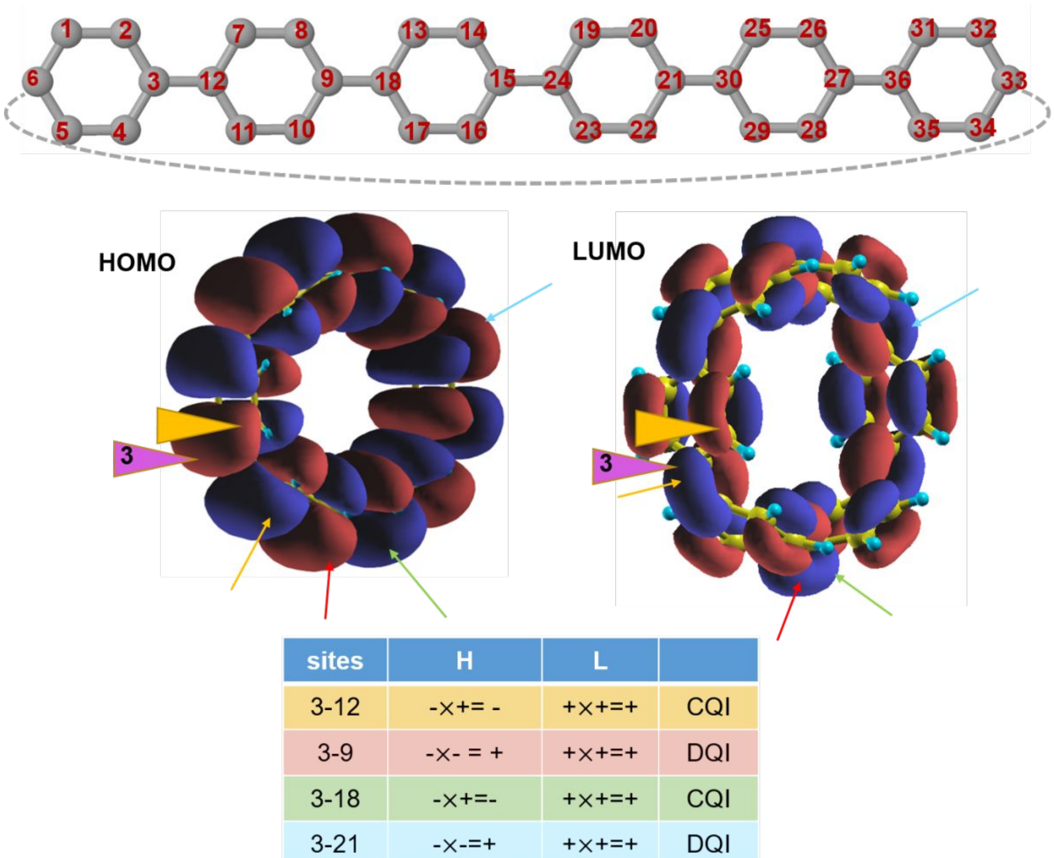

sites	H	L	
3-12	-x+= -	+x+=+	CQI
3-9	-x- = +	+x+=+	DQI
3-18	-x+=-	+x+=+	CQI
3-21	-x-=+	+x+=+	DQI

Figure S2. Molecular orbitals of CPP6. The second column of the table shows the HOMO product. The third column shows the LUMO product. The fourth column shows the product-rule prediction.

Figure S2 shows the highest occupied molecular orbital (HOMO) and lowest unoccupied molecular orbital (LUMO) of CPP6, in which blue regions depict orbital amplitudes of opposite sign to those of red regions. A simple orbital symmetry rule[2] predicts that if the products of the HOMO amplitudes at the source and drain have the same sign as the products of the LUMO amplitudes at the source and drain, then DQI occurs and the electrical conductance should be

low. Otherwise, if the HOMO and LUMO products have different signs, then CQI occurs and the electrical conductance should be high.

S3. Magic-number theory and tight-binding transmission calculations of CPP6: application of the magic ratio rule (MRR).

In the following sections and appendices, we provide a detailed analytical theory of transport properties based on tight-binding theory. The connectivity table for CPP6 is shown below:

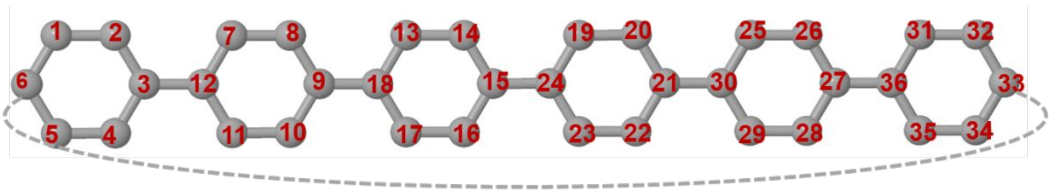

$\bar{C}^t_{ij}$	2	4	6	8	10	12	14	16	18	20	22	24	26	28	30	32	34	36
1	1	0	1	0	0	0	0	0	0	0	0	0	0	0	0	0	0	0
3	1	1	0	0	0	1	0	0	0	0	0	0	0	0	0	0	0	0
5	0	1	1	0	0	0	0	0	0	0	0	0	0	0	0	0	0	0
7	0	0	0	1	0	1	0	0	0	0	0	0	0	0	0	0	0	0
9	0	0	0	1	1	0	0	0	1	0	0	0	0	0	0	0	0	0
11	0	0	0	0	1	1	0	0	0	0	0	0	0	0	0	0	0	0
13	0	0	0	0	0	0	1	0	1	0	0	0	0	0	0	0	0	0
15	0	0	0	0	0	0	1	1	0	0	0	1	0	0	0	0	0	0
17	0	0	0	0	0	0	0	1	1	0	0	0	0	0	0	0	0	0
19	0	0	0	0	0	0	0	0	0	1	0	1	0	0	0	0	0	0
21	0	0	0	0	0	0	0	0	0	1	1	0	0	0	1	0	0	0
23	0	0	0	0	0	0	0	0	0	0	1	1	0	0	0	0	0	0
25	0	0	0	0	0	0	0	0	0	0	0	0	1	0	1	0	0	0
27	0	0	0	0	0	0	0	0	0	0	0	0	1	1	0	0	0	1
29	0	0	0	0	0	0	0	0	0	0	0	0	0	1	1	0	0	0
31	0	0	0	0	0	0	0	0	0	0	0	0	0	0	0	1	0	1
33	0	0	1	0	0	0	0	0	0	0	0	0	0	0	0	1	1	0
35	0	0	0	0	0	0	0	0	0	0	0	0	0	0	0	0	1	1

Figure S3. The connectivity table for CPP6.

The corresponding magic number table (which is proportional to the $E = 0$ Green's function obtained by inverting the connectivity table) is shown below:

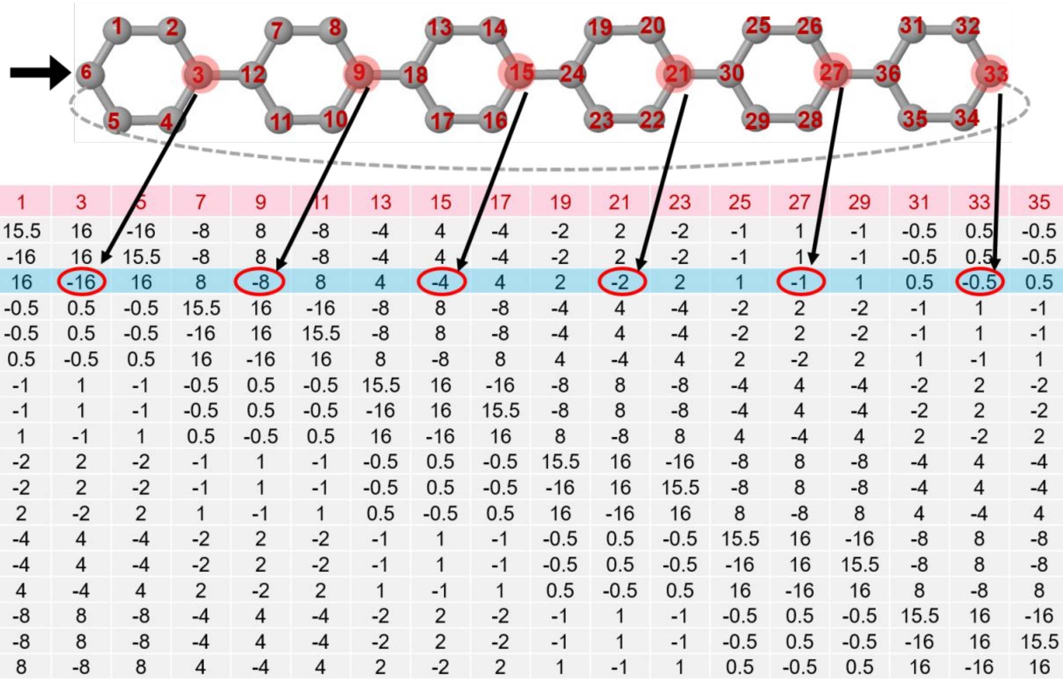

M_{ji}	1	3	5	7	9	11	13	15	17	19	21	23	25	27	29	31	33	35
2	15.5	16	-16	-8	8	-8	-4	4	-4	-2	2	-2	-1	1	-1	-0.5	0.5	-0.5
4	-16	16	15.5	-8	8	-8	-4	4	-4	-2	2	-2	-1	1	-1	-0.5	0.5	-0.5
6	16	-16	16	8	-8	8	4	-4	4	2	-2	2	1	-1	1	0.5	-0.5	0.5
8	-0.5	0.5	-0.5	15.5	16	-16	-8	8	-8	-4	4	-4	-2	2	-2	-1	1	-1
10	-0.5	0.5	-0.5	-16	16	15.5	-8	8	-8	-4	4	-4	-2	2	-2	-1	1	-1
12	0.5	-0.5	0.5	16	-16	16	8	-8	8	4	-4	4	2	-2	2	1	-1	1
14	-1	1	-1	-0.5	0.5	-0.5	15.5	16	-16	-8	8	-8	-4	4	-4	-2	2	-2
16	-1	1	-1	-0.5	0.5	-0.5	-16	16	15.5	-8	8	-8	-4	4	-4	-2	2	-2
18	1	-1	1	0.5	-0.5	0.5	16	-16	16	8	-8	8	4	-4	4	2	-2	2
20	-2	2	-2	-1	1	-1	-0.5	0.5	-0.5	15.5	16	-16	-8	8	-8	-4	4	-4
22	-2	2	-2	-1	1	-1	-0.5	0.5	-0.5	-16	16	15.5	-8	8	-8	-4	4	-4
24	2	-2	2	1	-1	1	0.5	-0.5	0.5	16	-16	16	8	-8	8	4	-4	4
26	-4	4	-4	-2	2	-2	-1	1	-1	-0.5	0.5	-0.5	15.5	16	-16	-8	8	-8
28	-4	4	-4	-2	2	-2	-1	1	-1	-0.5	0.5	-0.5	-16	16	15.5	-8	8	-8
30	4	-4	4	2	-2	2	1	-1	1	0.5	-0.5	0.5	16	-16	16	8	-8	8
32	-8	8	-8	-4	4	-4	-2	2	-2	-1	1	-1	-0.5	0.5	-0.5	15.5	16	-16
34	-8	8	-8	-4	4	-4	-2	2	-2	-1	1	-1	-0.5	0.5	-0.5	-16	16	15.5
36	8	-8	8	4	-4	4	2	-2	2	1	-1	1	0.5	-0.5	0.5	16	-16	16

Figure S4 Magic number tables $\overline{M}$ for CQI connectivities in the 6-ring macrocycle.

For macrocycle in Figure S4, each phenyl ring is connected to two neighbours. For ring j, let the site connected to ring $j-1$ be denoted j_p and the site connected to ring $j+1$ be denoted j_q. These sites are referred to as 'nodal sites. ' For example, the nodal sites 3_p and 3_q of ring 3 are numbered 18 and 15. The magic number table $\overline{D} = M^2$, which connects even to even sites is shown below. The corresponding Green's function connecting even to even sites is proportional to $E\overline{D}$(see appendix H).

M^2	2	4	6	8	10	12	14	16	18	20	22	24	26	28	30	32	34	36
2	64	1	-33	17	17	-33	10	10	-18	8	8	-12	10	10	-12	17	17	-18
4	1	64	-33	17	17	-33	10	10	-18	8	8	-12	10	10	-12	17	17	-18
6	-33	-33	65	-18	-18	34	-12	-12	20	-12	-12	16	-18	-18	20	-33	-33	34
8	17	17	-18	64	1	-33	17	17	-33	10	10	-18	8	8	-12	10	10	-12
10	17	17	-18	1	64	-33	17	17	-33	10	10	-18	8	8	-12	10	10	-12
12	-33	-33	34	-33	-33	65	-18	-18	34	-12	-12	20	-12	-12	16	-18	-18	20
14	10	10	-12	17	17	-18	64	1	-33	17	17	-33	10	10	-18	8	8	-12
16	10	10	-12	17	17	-18	1	64	-33	17	17	-33	10	10	-18	8	8	-12
18	-18	-18	20	-33	-33	34	-33	-33	65	-18	-18	34	-12	-12	20	-12	-12	16
20	8	8	-12	10	10	-12	17	17	-18	64	1	-33	17	17	-33	10	10	-18
22	8	8	-12	10	10	-12	17	17	-18	1	64	-33	17	17	-33	10	10	-18
24	-12	-12	16	-18	-18	20	-33	-33	34	-33	-33	65	-18	-18	34	-12	-12	20
26	10	10	-18	8	8	-12	10	10	-12	17	17	-18	64	1	-33	17	17	-33
28	10	10	-18	8	8	-12	10	10	-12	17	17	-18	1	64	-33	17	17	-33
30	-12	-12	20	-12	-12	16	-18	-18	20	-33	-33	34	-33	-33	65	-18	-18	34
32	17	17	-33	10	10	-18	8	8	-12	10	10	-12	17	17	-18	64	1	-33
34	17	17	-33	10	10	-18	8	8	-12	10	10	-12	17	17	-18	1	64	-33
36	-18	-18	34	-12	-12	20	-12	-12	16	-18	-18	20	-33	-33	34	-33	-33	65

Figure S5 Magic number table $\overline{D} = M^2$, with matrix element $\overline{D}_{ij}$=$(M^2)_{ij}$ for DQI connectivities in the 6-ring macrocycle

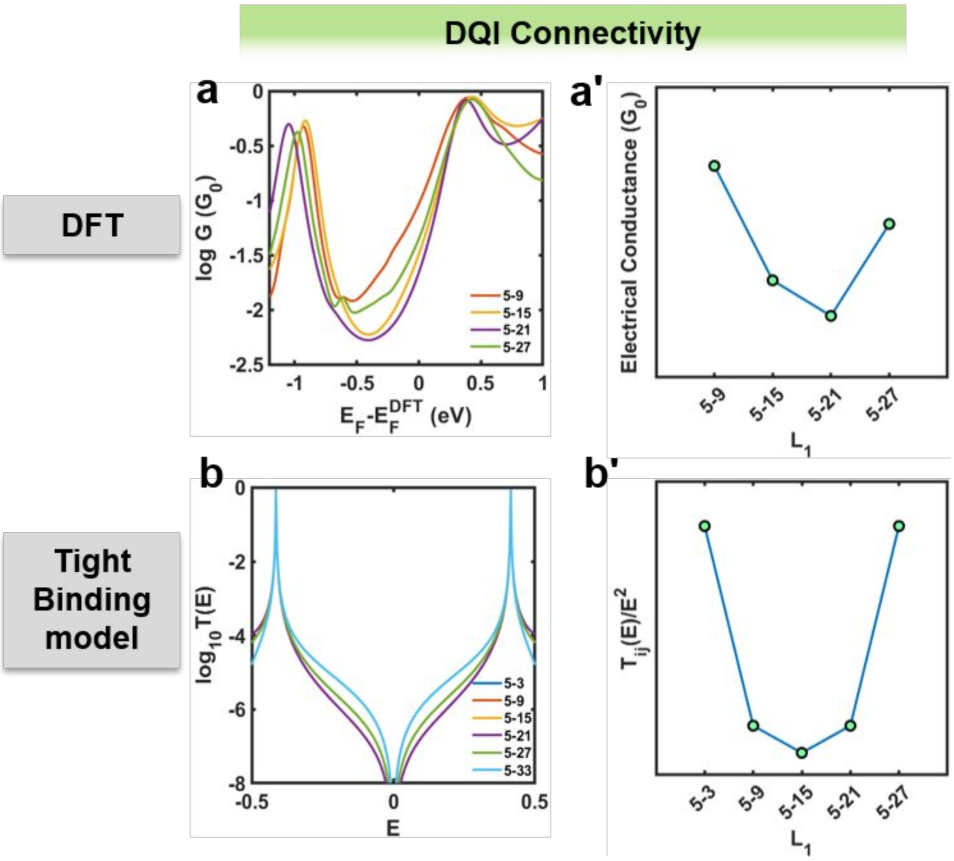

Figure S6. Transport properties for DQI CPP6 in the level of DFT and TBM with the left electrode fixed to site 5. (a, a') DQI electrical conductance as a function of the Fermi energy (E_F

) in units of the quantum conductance $G_0 = 77\mu S$ at room temperature $300K$ and mid-gap conductance of Figure S6a for different connectivity with length L_1. (b,b')DQI Transmission curves $T_{ij}(E)$ for a tight-binding model of CPP6 of Figure 3a and mid-gap transmission coefficients $T_{ij}(0)$ of Figure S6b. It is worth to mentioning that the connectivities 5-3 and 5-33 cannot be accessed in a DFT calculation, since the two electrodes are very close and would interact with each other directly.

S4. Transport properties of CPP11 obtained using both DFT and a tight-binding model:

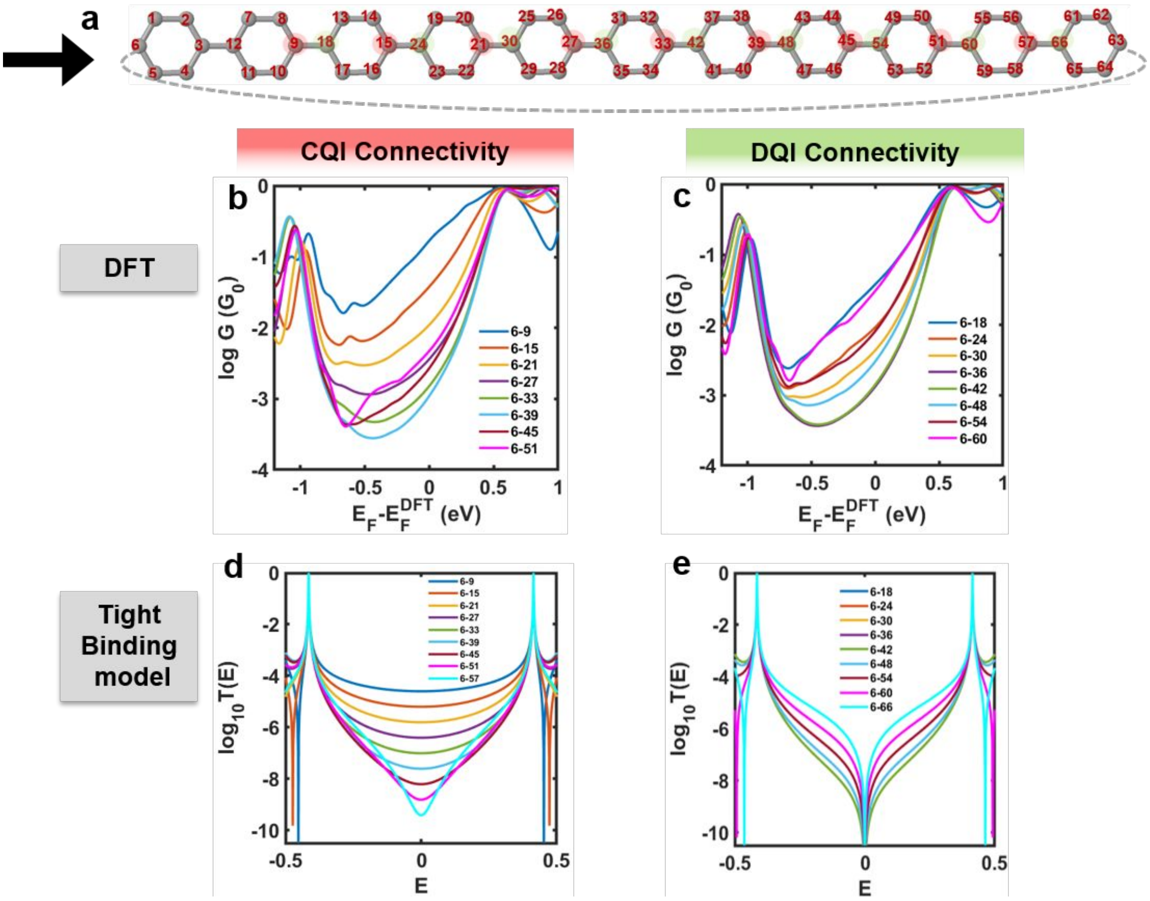

Figure S7. (a) A tight binding lattice representing a 11-ring macrocycle, showing the numbering system (b,c) CQI and DQI DFT-based electrical conductance as a function of the fermi energy at room temperature 300K. (d-e) CQI and DQI transmission curves $T_{ij}(E)$ for a tight-binding model.

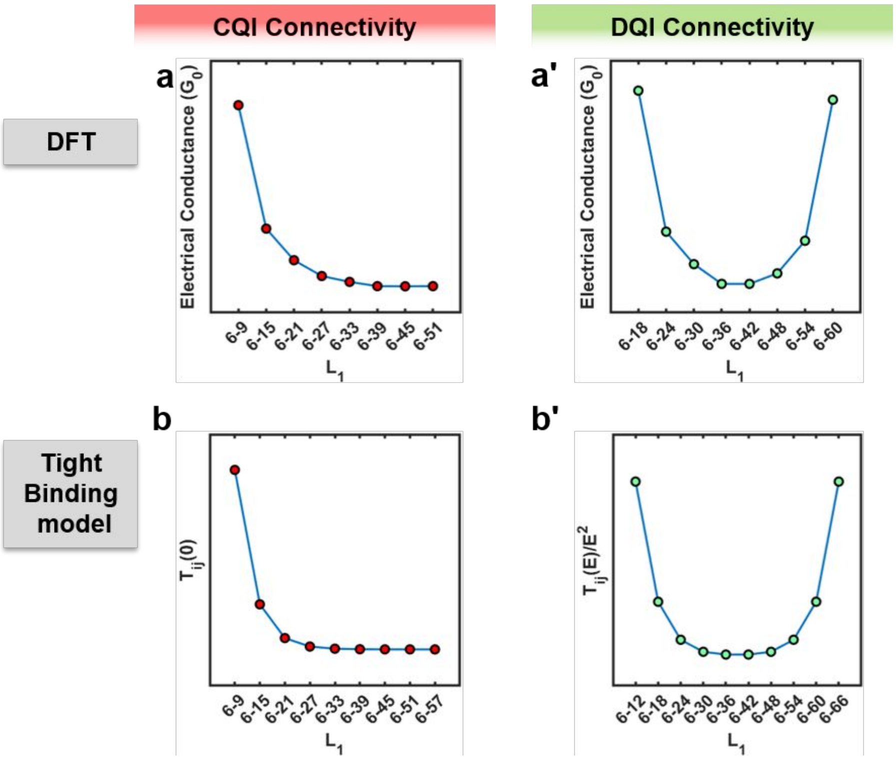

Figure S8a. (a and a') CQI and DQI mid-gap conductance for different connectivity with length $L_1$of Figure S7b,c . (b and b') CQI and DQI mid-gap transmission coefficients $T_{ij}(0)$ of Figure S7d and $T_{ij}(E)$ of Figure S7e versus the connectivity between sites i and j as long as 6-ring macrocycle length L_1.

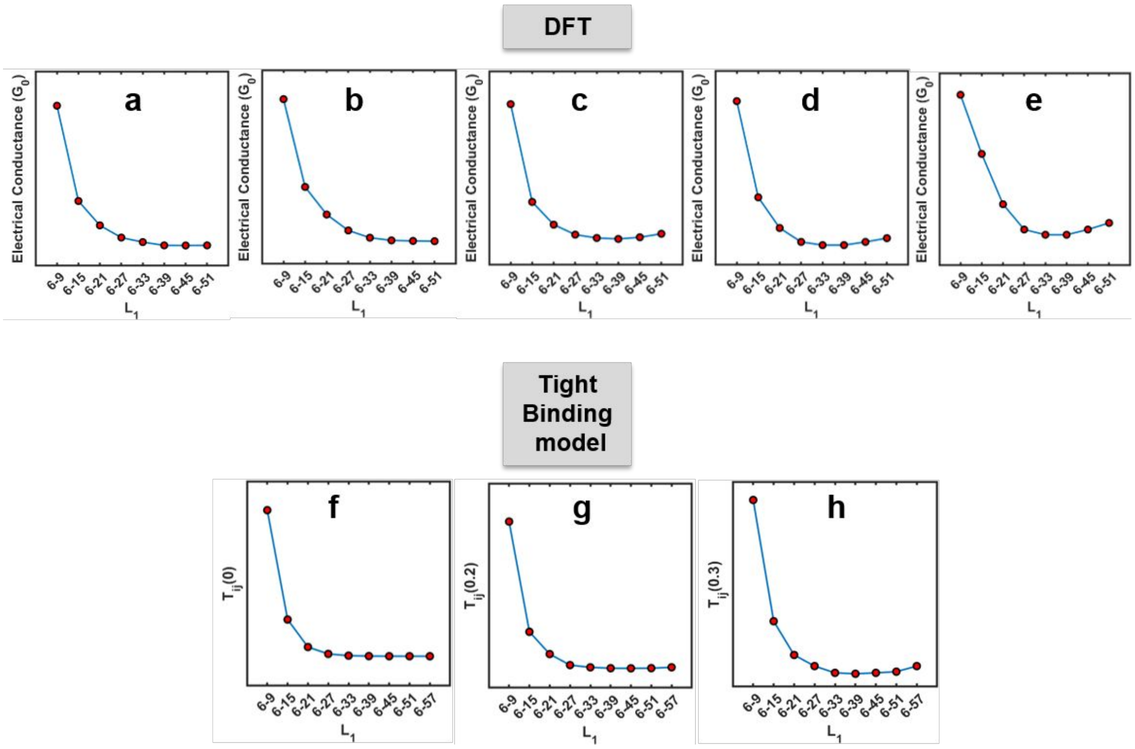

Figure S8b. (a-e) CQI mid-gap conductance for different connectivity at energy points around the middle of HOMO-LUMO gap figure S7b, e.g. -0.7, -0.66, -0.5, 0.25, 0.4 respectively, where E is the energy relative to the Fermi energy predicted by DFT. (f-h) CQI mid-gap transmission coefficients $T_{ij}(0)$ of figure S7d at energy points around the middle of HOMO-LUMO gap, e.g. 0, 0.2, and 0.3 respectively.

Based on the DFT-results, CPP11 shows that the electrical conductance first decreases and then increases. Modifying our simple TBM could help to give a qualitative behaviour of DFT results, by introducing second nearest neighbour coupling between the ring sites as shown in Figure S9a. After introducing this coupling the symmetry around the zero in Figure S7d disappears, as it shown in Figure S9b.

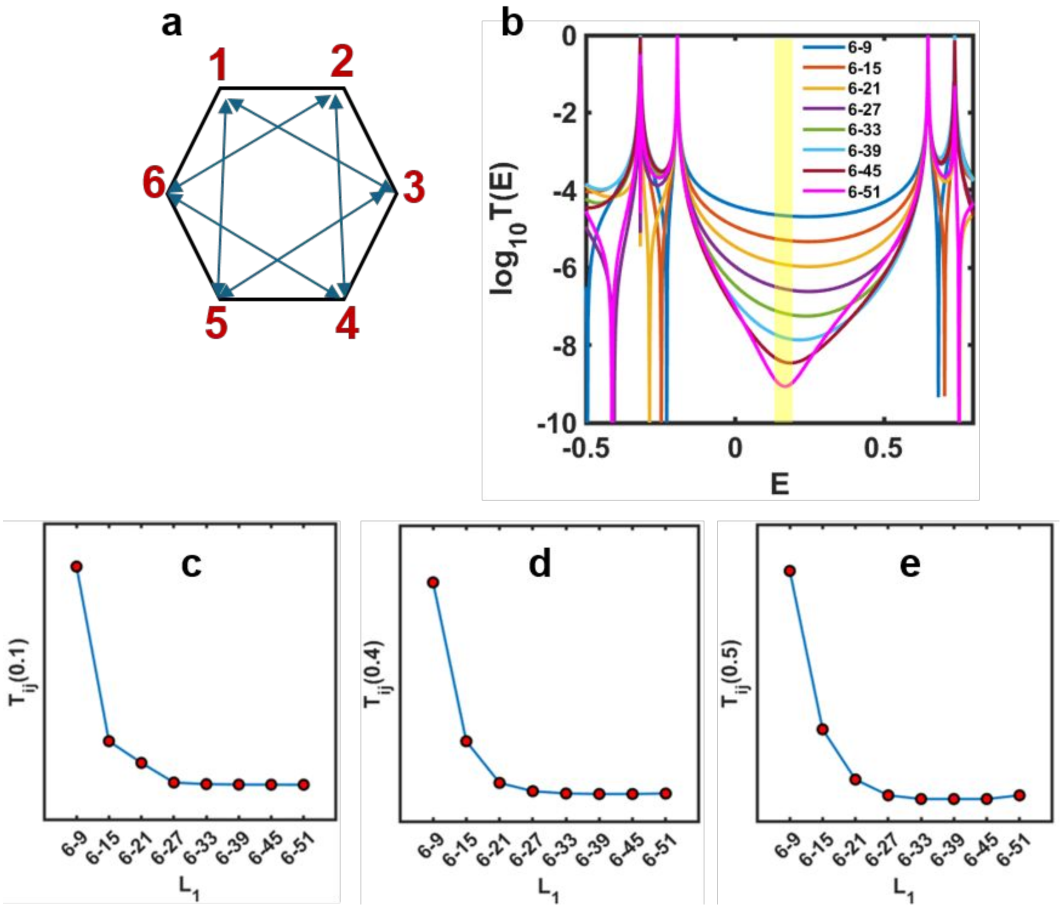

Figure S9. (a) A tight binding lattice representing a 1-ring macrocycle with 2nd nearest neighbour coupling (i.e site 1 connects to site 3 and site 2 connects to site 4), and the coupling bond is equal to -0.25 , (b) CQI transmission curves $T_{ij}(E)$ for a tight-binding model of the 11-ring macrocycle with 2nd nearest neighbour coupling for each ring. (c-e) CQI mid-gap transmission coefficients $T_{ij}(E)$ of figure S9b at energy points around the middle of HOMO-LUMO gap, e.g. 0.1, 0.4, and 0.5 respectively.

S5. Magic number theory of a linear chain of phenyl rings.

Magic-Number table of 6-phenyl-ring linear chain

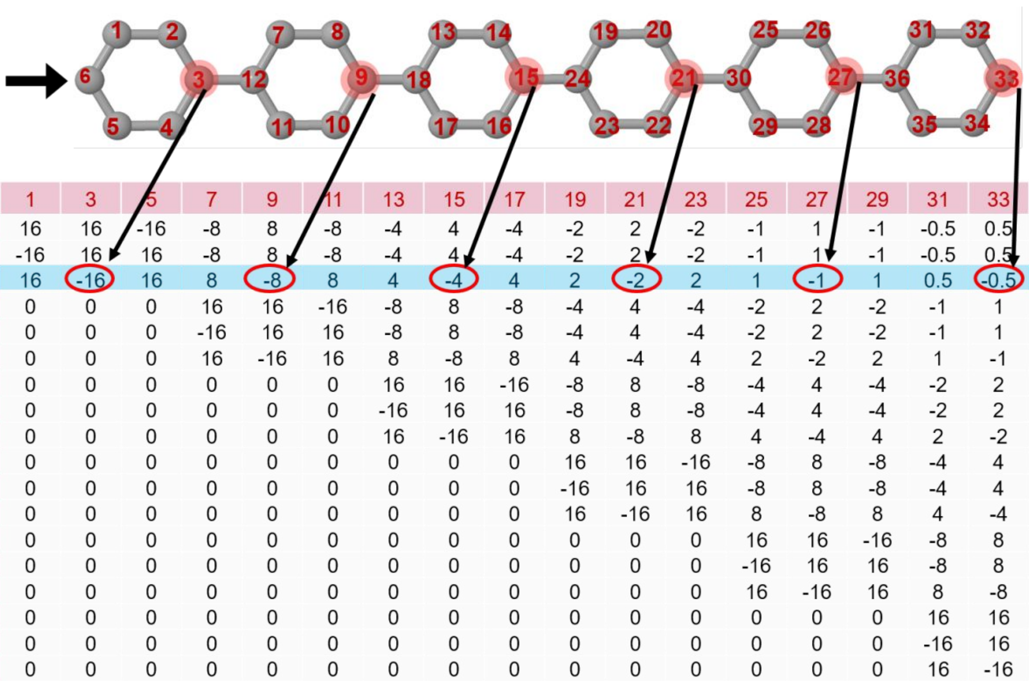

M_{ji}	1	3	5	7	9	11	13	15	17	19	21	23	25	27	29	31	33	35
2	16	16	-16	-8	8	-8	-4	4	-4	-2	2	-2	-1	1	-1	-0.5	0.5	-0.5
4	-16	16	16	-8	8	-8	-4	4	-4	-2	2	-2	-1	1	-1	-0.5	0.5	-0.5
6	16	-16	16	8	-8	8	4	-4	4	2	-2	2	1	-1	1	0.5	-0.5	0.5
8	0	0	0	16	16	-16	-8	8	-8	-4	4	-4	-2	2	-2	-1	1	-1
10	0	0	0	-16	16	16	-8	8	-8	-4	4	-4	-2	2	-2	-1	1	-1
12	0	0	0	16	-16	16	8	-8	8	4	-4	4	2	-2	2	1	-1	1
14	0	0	0	0	0	0	16	16	-16	-8	8	-8	-4	4	-4	-2	2	-2
16	0	0	0	0	0	0	-16	16	16	-8	8	-8	-4	4	-4	-2	2	-2
18	0	0	0	0	0	0	16	-16	16	8	-8	8	4	-4	4	2	-2	2
20	0	0	0	0	0	0	0	0	0	16	16	-16	-8	8	-8	-4	4	-4
22	0	0	0	0	0	0	0	0	0	-16	16	16	-8	8	-8	-4	4	-4
24	0	0	0	0	0	0	0	0	0	16	-16	16	8	-8	8	4	-4	4
26	0	0	0	0	0	0	0	0	0	0	0	0	16	16	-16	-8	8	-8
28	0	0	0	0	0	0	0	0	0	0	0	0	-16	16	16	-8	8	-8
30	0	0	0	0	0	0	0	0	0	0	0	0	16	-16	16	8	-8	8
32	0	0	0	0	0	0	0	0	0	0	0	0	0	0	0	16	16	-16
34	0	0	0	0	0	0	0	0	0	0	0	0	0	0	0	-16	16	16
36	0	0	0	0	0	0	0	0	0	0	0	0	0	0	0	16	-16	16

Figure S10. The magic number tables $\overline{M}$ for 6-ring linear chain.

Transmission coefficients of the above linear chain using both DFT and a tight-binding model:

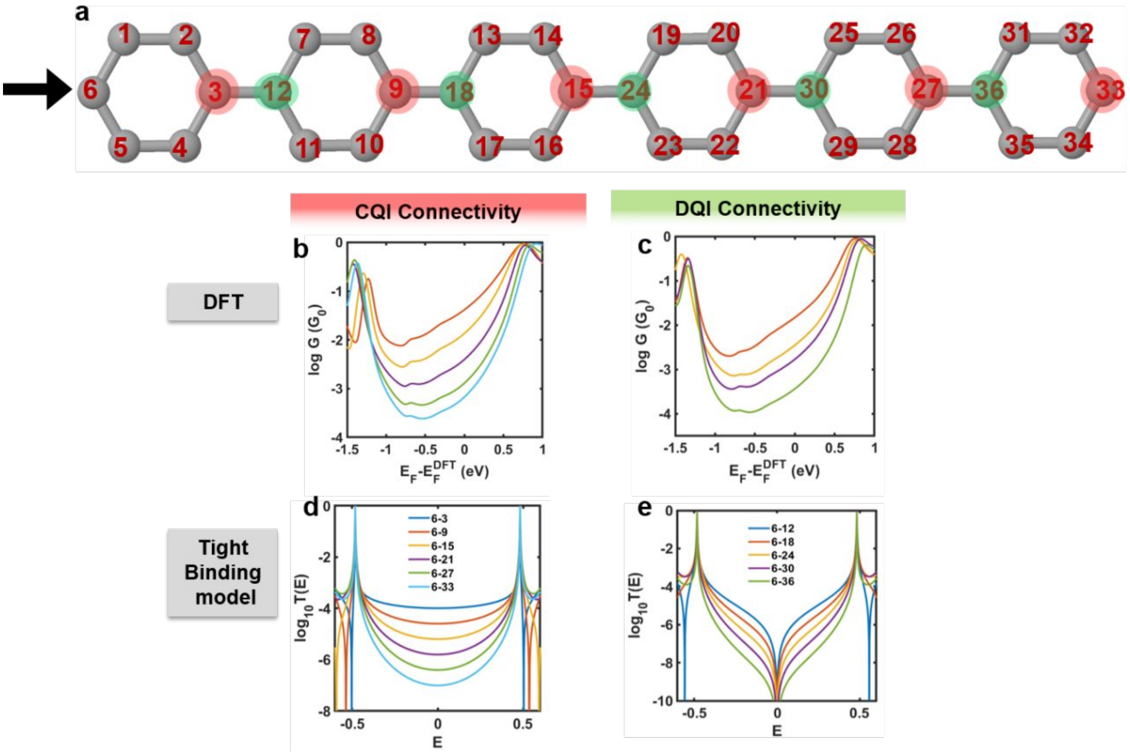

Figure S11. (a) A tight binding lattice representing a 6-ring linear chain cells, showing the numbering system. (b,c) CQI and DQI DFT-based electrical conductance as a function of the fermi energy at room temperature $300K$. (d,e) CQI and DQI transmission curves $T_{ij}(E)$ for a tight-binding model of the 6-ring linear chain cells.

S6. Theory of edge states in macrocycles.

In this section and in the appendices below, we present a derivation of the full mid-gap Green's function of an arbitrary macrocycle and note a few associated properties, some of which are known from the literature.

Calculation of Green's functions and magic number tables of CPP macrocycles

In general, one can regard each phenyl ring as an example of cell of tight-binding sites. In what follows, for a macrocycle of N cells with periodic boundary conditions or a linear chain of N cells, sites are labelled $(i)_r$, where $i = 1,2,\ldots N$. For example, for cells composed of 6-membered rings, $r = 1,2\ldots6$. Sites which connect to neighbouring cells will be referred to as 'nodal sites'. In what follows, the two nodal sites on cell j are labelled $(j)_p$, $(j)_q$ and the Green's function amplitudes on these sites will be denoted ϕ_j and ψ_j. For example for the following structure, $p = 1$ and $q = 4$:

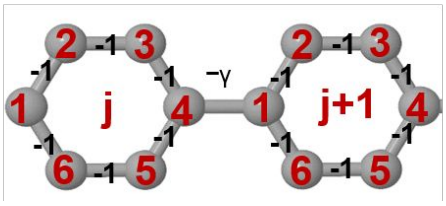

Figure S12: Two phenyl rings, with couplings between adjacent nodal sites $(j)_4$ and $(J+1)_1$. The Hamiltonian matrix element between neighbouring sites within the same cell will be denoted $-\gamma'$. The Hamiltonian matrix element between neighbouring nodal sites on neighbouring cells will be denoted $-\gamma$. In what follows, we shall choose $\gamma' = 1$.

Each of the above phenyl rings is an example of an arbitrary closed structure, which is represented by a rectangle in Figure S13 below:

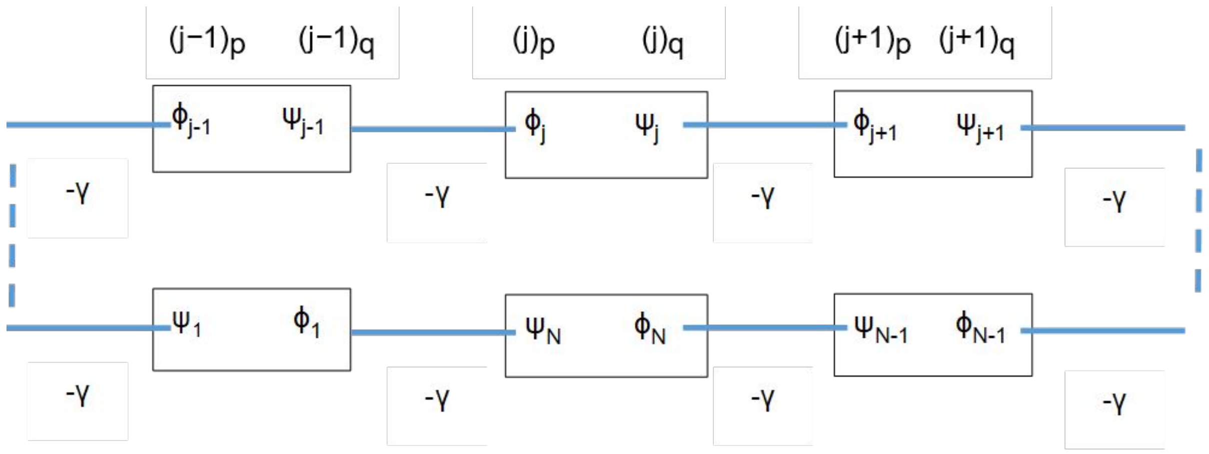

Figure S13. A macrocycle of N cells with periodic boundary conditions.

Note that for a macrocycle, or a linear chain of N cells, the Green's function is symmetric; ie $G_{(j)_q(l)_p} = G_{(l)_p(j)_q}$. On the other hand, $G_{(j)_q(l)_p} \neq G_{(j)_p(l)_q}$ which means that the wave on sub-lattice q, created by a source on sub-lattice p is not the same as the wave on sub-lattice p created by a source on sub-lattice q. By symmetry, one also expects that for a macrocycle, $G_{(j)_p(l)_{p'}} = G_{(j)_q(l)_q}$, which means that the wave on sub-lattice p created by a source on sub-lattice p is the same as the wave on sub-lattice q created by a source on sub-lattice q. On the other hand for a linear chain, one expects $G_{(j)_p(l)_p} = G_{(N+1-j)_q(N+1-l)_q}$. These will be derived explicitly below.

To obtain the Green's function amplitudes on all nodal sites, it is convenient to first decimate all non-nodal sites, to obtain a renormalised, energy-dependent 2x2 Hamiltonian $\bar{h}$, describing the intra-cell couplings of each cell, as shown in Figure S15 of section7.

The Green's function G due to a source acting on the nodal site $(l)_p$ (ie acting on site p of cell l) has matrix elements on all nodal sites of the form

$\begin{pmatrix} G_{(j)_p(l)_p} \\ G_{(j)_q(l)_p} \end{pmatrix} = \begin{pmatrix} \phi_j \\ \psi_j \end{pmatrix}$ and satisfies

$$(E - \overline{h})\begin{pmatrix} \phi_j \\ \psi_j \end{pmatrix} = h_{-1}\begin{pmatrix} \phi_{j-1} \\ \psi_{j-1} \end{pmatrix} + h_1\begin{pmatrix} \phi_{j+1} \\ \psi_{j+1} \end{pmatrix} + \begin{pmatrix} 1 \\ 0 \end{pmatrix}\delta_{jl}$$

As discussed in the textbook [9], as an alternative to decimation, the renormalised intra-cell Hamiltonian can also be obtained from $(E - \overline{h}) = \overline{g}^{-1}$, where $\overline{g}$ is the $2x2$ matrix containing Green's function matrix elements of an isolated cell, involving only the two nodal sites of the cells. Since the inter-cell couplings γ are not affected by decimating non-nodal sites, we are interested in the case where

$$(E - \overline{h}) = \begin{pmatrix} \epsilon & \alpha \\ \alpha & \epsilon \end{pmatrix}, \quad h_1 = -\gamma\begin{pmatrix} 0 & 0 \\ 1 & 0 \end{pmatrix} \text{ and } h_{-1} = h_1^{\dagger} = -\gamma\begin{pmatrix} 0 & 1 \\ 0 & 0 \end{pmatrix}$$

The intra-cell parameters (α)will be obtained later. The equation describing the Green's function of nodal sites then takes the following form:

$$\begin{pmatrix} \epsilon\phi_j + \alpha\psi_j \\ \epsilon\psi_j + \alpha\phi_j \end{pmatrix} = -\gamma\begin{pmatrix} \psi_{j-1} \\ \phi_{j+1} \end{pmatrix} + \begin{pmatrix} 1 \\ 0 \end{pmatrix}\delta_{jl} \quad (1)$$

For $j \neq l$, the solution must be a linear superposition of plane waves of the form

$$\begin{pmatrix} \phi_j' \\ \psi_j' \end{pmatrix} = \begin{pmatrix} \phi \\ \psi \end{pmatrix} e^{ikj} \quad (2)$$

where

$$\begin{pmatrix} \epsilon\phi + \alpha\psi + \gamma\psi e^{-ik} \\ \epsilon\psi + \alpha\phi + \gamma\phi e^{ik} \end{pmatrix} = \begin{pmatrix} 0 \\ 0 \end{pmatrix} \qquad (3)$$

Ie

$$\begin{pmatrix} \epsilon & \Delta(k) \\ \Delta^*(k) & \epsilon \end{pmatrix} \begin{pmatrix} \phi \\ \psi \end{pmatrix} = \begin{pmatrix} 0 \\ 0 \end{pmatrix} \qquad (4)$$

where

$$\Delta(\mathrm{k}) = \alpha + \gamma e^{-ik} \qquad (5)$$

As an example, to compute the Green's function at the centre of the HOMO-LUMO gap, consider the case $\epsilon = 0$. This case, equation (3) yields

$$\psi(\alpha + \gamma e^{-ik}) = 0 \quad \text{and} \quad \phi(\alpha + \gamma e^{ik}) = 0 \qquad (6)$$

Hence either

$$e^{ik} = -\gamma/\alpha \quad \text{and} \quad \phi = 0 \text{ or } \quad e^{-ik} = -\gamma/\alpha \quad \text{and} \quad \psi = 0 \qquad (7)$$

Define the real number β by

$$e^{\beta} = |\frac{\gamma}{\alpha}| \qquad (8)$$

and also define the sign s by $s = 1$ if $\alpha\gamma > 0$ and $s = 0$ if $\alpha\gamma < 0$. (9)

Then

$$k = i\beta + \pi s \qquad (10)$$

$$e^{ik} = e^{-\beta} e^{i\pi s}$$

$$e^{i\pi s} = e^{-i\pi s} = \pm 1 = (-1)^s$$

and equation (2) yields two possible solutions:

$$\begin{pmatrix}\phi'_j\\ \psi'_j\end{pmatrix} = A\begin{pmatrix}1\\0\end{pmatrix}e^{-\beta j}e^{i\pi sj} \quad \text{or} \quad \begin{pmatrix}\phi'_j\\ \psi'_j\end{pmatrix} = B\begin{pmatrix}0\\1\end{pmatrix}e^{\beta j}e^{i\pi sj} \tag{11}$$

where A and B are constants, which do not depend on j.

The Green's function taking into account the source at site $(l)_p$, must therefore have the form

$$\begin{pmatrix}\phi_j\\ \psi_j\end{pmatrix} = \frac{1}{\alpha}\left[\begin{pmatrix}0\\1\end{pmatrix}e^{\beta(j-l)}e^{i\pi s(j-l)}\Theta(j-l) + a\begin{pmatrix}0\\1\end{pmatrix}e^{\beta(j-l)}e^{i\pi s(j-l)} + b\begin{pmatrix}1\\0\end{pmatrix}e^{-\beta(j-l)}e^{i\pi s(j-l)}\right] \tag{12}$$

where a and b are constants and $\Theta(j-l)$ is a step function defined by

$$\Theta(j-l) = 1 \text{ for } j \geq l \qquad \text{and} \qquad \Theta(j-l) = 0 \text{ for } j < l. \tag{13}$$

For $j = l$, equation (1) yields (since $\epsilon = 0$),

$$\gamma\psi_{l-1} + \alpha\psi_l = 1 \qquad \text{and} \qquad \gamma\phi_{l+1} + \alpha\phi_l = 0 \tag{14}$$

Substituting the expression (12) into these shows that equations (14) are satisfied for any choice a and b. On the other hand, from Figure S13, periodic boundary conditions require

$$\phi_1 = \phi_{N+1} \quad \text{and} \quad \psi_0 = \psi_N \tag{15a}$$

Substituting the expression (12) into these shows that the first of equations (15) can only be satisfied if $b = 0$ and therefore

$$\begin{pmatrix}G_{(j)_p(l)_p}\\ G_{(j)_q(l)_p}\end{pmatrix} = \frac{1}{\alpha}\begin{pmatrix}0\\1\end{pmatrix}e^{\beta(j-l)}e^{i\pi s(j-l)}[\Theta(j-l) + a] \qquad \text{(periodic chain)} \tag{16a}$$

Substituting the expression (16) into second of equations (15) yields

$$e^{\beta(N-l)}e^{i\pi s(N-l)}[1+a] = ae^{-\beta l}e^{-i\pi sl)}$$

Ie $$a = \frac{e^{\beta N}e^{i\pi sN}}{(1-e^{\beta N}e^{i\pi sN})}$$

Similarly for a source at site $(l)_q$ (see appendix A),

$$\begin{pmatrix} G_{(j)_p(l)_q} \\ G_{(j)_q(l)_q} \end{pmatrix} = \frac{1}{\alpha}\begin{pmatrix} 1 \\ 0 \end{pmatrix} e^{-\beta(j-l)}e^{i\pi s(j-l)}[\Theta(l-j)+a] \qquad \text{(periodic chain)} \qquad (16b)$$

Equations (16a) and (16b) are equations (3) and (5) of the main text, for the Green's function of a periodic chain. On the other hand for a chain with free ends, the boundary conditions (15) are replaced by

$$\phi_{N+1} = 0 \qquad \text{and} \qquad \psi_0 = 0 \qquad (15b)$$

Substituting equation (12) into these yields $a = b = 0$ and hence the Green's function of a linear chain is

$$\begin{pmatrix} G_{(j)_p(l)_p} \\ G_{(j)_q(l)_p} \end{pmatrix} = \frac{1}{\alpha}\begin{pmatrix} 0 \\ 1 \end{pmatrix} e^{\beta(j-l)}e^{i\pi s(j-l)}\Theta(j-l) \qquad \text{(linear chain)} \quad (17.a)$$

Similarly for a source at site $(l)_q$ (see appendix A),

$$\begin{pmatrix} G_{(j)_p(l)_q} \\ G_{(j)_q(l)_q} \end{pmatrix} = \frac{1}{\alpha}\begin{pmatrix} 1 \\ 0 \end{pmatrix} e^{-\beta(j-l)}e^{i\pi s(j-l)}\Theta(l-j) \quad \text{(linear chain)} \quad (17.b)$$

Note that in both cases, the Green's function is symmetric; ie $G_{(j)_q(l)_p} = G_{(l)_p(j)_q}$. On the other hand, $G_{(j)_q(l)_p} \neq G_{(j)_p(l)_q}$, which means that the wave on sub-lattice q, created by a source on sub-lattice p is not the same as the wave on sub-lattice p created by a source on sub-lattice q.

The Greens function and renormalised Hamiltonian of a phenyl ring.

As discussed in section 10.8 of ref [3] the Green's function of an isolated ring of 6 sites of the form shown in Figure S12 is

$$\hat{g}_{rs} = C cos\, k(|r - s| - 3) \qquad \text{where} \quad C = \frac{1}{2\gamma' \sin k \sin 3k} \qquad (18)$$

where k is an energy-dependent wave vector and γ' is the nearest neighbour coupling within the ring. (In figure S12, $\gamma' = 1$ and $k = \cos^{-1} E/2\gamma'$). In the above expression, r and s label any of the 6 sites within the ring. At the gap centre, $k = \frac{\pi}{2}$ and therefore the mid-gap Green's function of a phenyl ring is

$$\hat{g}_{rr'} = \frac{1}{2\gamma'} sin \frac{\pi}{2}(|r - s|) \quad \text{(mid-gap phenyl ring)} \qquad (19)$$

Hence $\hat{g}_{rr} = 0$. Similarly for meta-connected sites: $\hat{g}_{r,r\pm2} = 0$, for ortho-connected sites: $\hat{g}_{r,r\pm1} = \frac{1}{2\gamma'}$ and for para-connected sites: $\hat{g}_{r,r\pm3} = \frac{-1}{2\gamma'}$. Hence the 2x2 Green's function formed from elements involving sites $p = 1$ and $q = 4$ (which are para-connected) is

$$\bar{g} = \begin{pmatrix} 0 & -1/2\gamma' \\ -1/2\gamma' & 0 \end{pmatrix} \qquad (20)$$

This yields

$$\bar{g}^{-1} = \begin{pmatrix} 0 & -2\gamma' \\ -2\gamma' & 0 \end{pmatrix} \qquad (21)$$

As noted above equation (1), $(E - \bar{h}) = \bar{g}^{-1}$. Therefore

$$(E - \bar{h}) = \begin{pmatrix} \epsilon & \alpha \\ \alpha & \epsilon \end{pmatrix} = \begin{pmatrix} 0 & -2\gamma' \\ -2\gamma' & 0 \end{pmatrix} \qquad (22)$$

Hence at the mid-gap, $\alpha = -2\gamma'$ and $\epsilon = 0$. The same result could be obtained by decimating the phenyl ring at $E = 0$, to eliminate sites 2,3,5 and 6, as described in section 12.2 of ref [3].

Example $\boldsymbol{\alpha = -2\gamma, \epsilon = 0}$

In this case, $\alpha\gamma < 0$, so the sign $s = 0$. Also $e^{\beta} = 1/2$, so from equation (16), choosing $p = 1$ and $q = 4$ yields

$$\begin{pmatrix} G_{(j)_1(l)_1} \\ G_{(j)_4(l)_1} \end{pmatrix} = \begin{pmatrix} \phi_j \\ \psi_j \end{pmatrix} = \frac{-1}{2\gamma}\begin{pmatrix} 0 \\ 1 \end{pmatrix} 2^{-(j-l)}[\Theta(j-l) + a] \quad \text{(periodic chain)} \qquad (23a)$$

$$\begin{pmatrix} G_{(j)_1(l)_4} \\ G_{(j)_4(l)_4} \end{pmatrix} = \frac{-1}{2\gamma}\begin{pmatrix} 1 \\ 0 \end{pmatrix} 2^{(j-l)}[\Theta(l-j) + a] \qquad \text{(periodic chain)} \qquad (23b)$$

$$\begin{pmatrix} G_{(j)_1(l)_1} \\ G_{(j)_4(l)_1} \end{pmatrix} = \begin{pmatrix} \phi_j \\ \psi_j \end{pmatrix} = \frac{-1}{2\gamma}\begin{pmatrix} 0 \\ 1 \end{pmatrix} 2^{-(j-l)}\Theta(j-l) \qquad \text{(linear chain)} \qquad (24a)$$

$$\begin{pmatrix} G_{(j)_1(l)_4} \\ G_{(j)_4(l)_4} \end{pmatrix} = \frac{-1}{2\gamma}\begin{pmatrix} 1 \\ 0 \end{pmatrix} 2^{(j-l)}\Theta(l-j) \qquad \text{(linear chain)} \qquad (24b)$$

This completes the calculation for the 2x2 matrix

$$\bar{G}_{jl} = \begin{pmatrix} G_{(j)_1(l)_1} & G_{(j)_1(l)_4} \\ G_{(j)_4(l)_1} & G_{(j)_4(l)_4} \end{pmatrix} \qquad (25)$$

The above expressions yield Green's function elements involving sites $(j)_1$ and $(j)_4$, due to a source at site $(l)_1$ or $(l)_4$ They are the sub-blocks of a $2Nx2N$ matrix denoted G_{11}, which contains matrix elements between all nodal sites $(j)_p$ and $(l)_q$, with $j,l = 1,2, ..N$ and $p,q = 1,4$. In contrast, the complete Green's function of the structure in figure S13 is a $6Nx6N$ matrix denoted G. Therefore our next task is to use the above expression for the $2Nx2N$ matrix G_{11} to obtain an expression for the $6Nx6N$ matrix G. As shown in appendix B, these are given by

$$G_{(j)_1(l)_2} = \frac{1}{2\gamma}\delta_{jl} - \frac{1}{2}G_{(j)_1(l-1)_4} \qquad (26)$$

$$G_{(j)_2(l)_3} = \frac{1}{2\gamma}[\delta_{jl} - \frac{1}{2}\delta_{j-1,l}] + \frac{1}{4}G_{(j-1)_4(l+1)_1} \qquad (27)$$

$$G_{(j)_3(l)_4} = \frac{1}{2\gamma}\delta_{jl} - \frac{1}{2}G_{(j+1)_1(l)_4} \qquad (28)$$

Note that by symmetry $G_{(l)_2(j)_1} = G_{(j)_1(l)_2}$ and $G_{(j)_2(l)_3} = G_{(l)_3(j)_2}$. Furthermore, for periodic boundary conditions, $G_{(j)_3(j+l)_4} = G_{(j)_2(j-l)_1}$.

As examples, appendix B shows that for periodic boundary conditions $G_{(j)_3(j)_4} = G_{(j)_1(j)_2} = \frac{1}{2\gamma}[1 + a]$, whereas $G_{(j)_2(j)_3} = G_{(j)_3(j)_2} = \frac{1}{2\gamma}[1 - a]$. Hence the ratio $\frac{G_{(j)_3(j)_2}}{G_{(j)_3(j)_4}}$ becomes

$$\frac{G_{(j)_3(j)_2}}{G_{(j)_3(j)_4}} = 1 - \frac{1}{2^{N-1}}$$

In contrast, for a linear chain, $G_{(j)_3(j)_4} = G_{(j)_1(j)_2} = \frac{1}{2\gamma}$, $G_{(j)_3(j)_2} = 0$

S7. The topological origin of the non-classical conductance behaviour of CPP macrocycles.

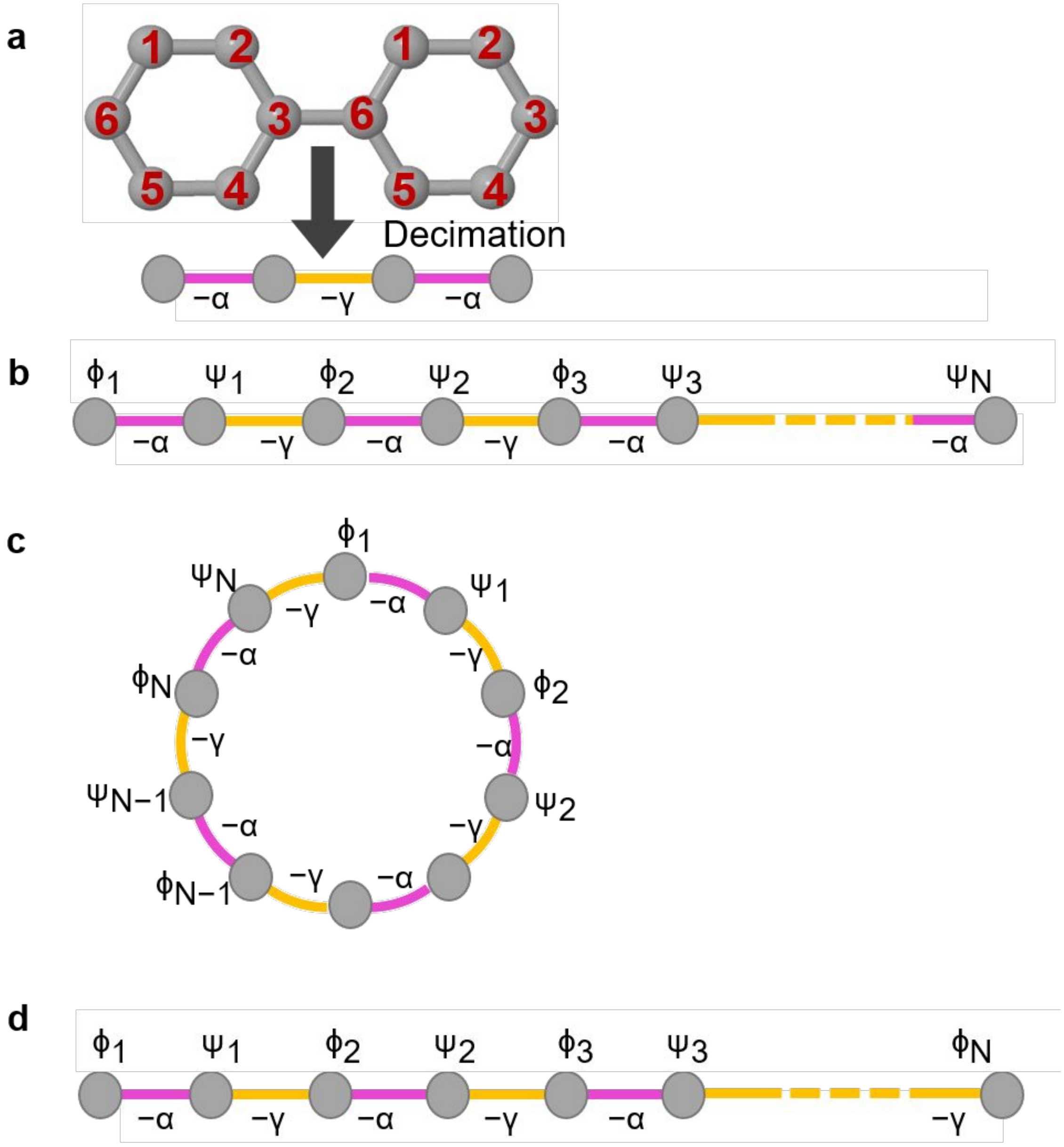

Figure S14. Schemes for tight binding model of a macrocycle onto a linear chain of alternating bonds (a) A TBM for two rings of a larger macrocycle. All sites have nearest neighbour couplings equal to $-\gamma$, where $\gamma = 1$. At $E = 0$, after decimating non-nodal sites one obtains a

renormalised Hamiltonian containing only nodal sites, with alternating bonds $-\alpha$ and $-\gamma$. For $\gamma = 1$, one obtains $\alpha = -2$, so that $\frac{\alpha}{\gamma} = -2$. (b) A linear chain of alternating bonds, obtained by decimating a linear chain of N phenyl rings. (c) A chain of alternating bonds with periodic boundary conditions, obtained by decimating a tight-binding model of a macrocycle containing N phenyl rings. (d) A linear chain of alternating bonds terminated with the different bonds at opposite ends.

In the latter case, for $N \rightarrow \infty$, if the quantities ψ_j and ϕ_j denote the amplitudes of an eigenstate at $E = 0$, then one finds $\phi_j = 0$ for all j, and

$$\psi_j \propto \left(\frac{1}{2}\right)^{(N-j)} \tag{29}$$

More details of the properties of a linear chain of phenyl rings are presented in S6 section. Obviously the chain with periodic boundary conditions in Figure S14c, which represents a macrocycle, contains no edges and therefore no edge states. Nevertheless, one can show that edge states play a crucial role in determining the non-classical conductance behaviour described above, because an edge state appears when an electrode is attached to the macrocycle. To illustrate this point, we examine the Green's function of the macrocycle. For a source located on the left nodal site of phenyl ring l, as shown in Figure S15a, the ($E = 0$) Green's function amplitudes on all other sites are denoted ϕ_j and ψ_j. Since this is a bipartite lattice,

magic number theory[3,4–6] tells us that $\phi_j = 0$ for all j. At $E = 0$, one finds that the Green's function elements ψ_j vanish on the left, (ie. $\psi_j = 0$ for $j < l$), while on the right they take the form

$$\psi_j \propto \left(\frac{1}{2}\right)^{(j-l)} \quad \text{for } j \geq l \qquad (30)$$

Intuitively, this means that at $E = 0$, the Green's function equations of Figure S16a are identical to those of Figure S15b, which contains two edges. Since $|\gamma| < |\alpha|$, as $N \rightarrow \infty$, an edge state is present on the right and no edge state is present on the left. For a source connected to the left nodal site of phenyl ring l and a drain connected to the right nodal site of ring j, the electrical conductance is proportional to $|\psi_j|^2$ and therefore decays exponential with the distance $|j - l|$ for for $j \geq l$, whereas the conductance is suppressed for $j < l$. This non-classical behaviour is a signature of topological edge states and is a manifestation of destructive quantum interference.

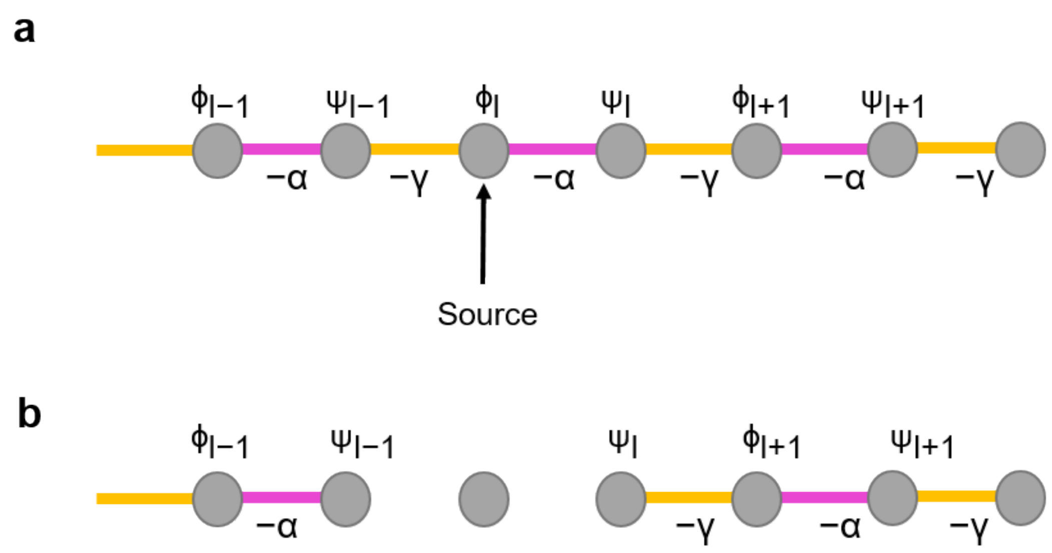

Figure S15. Single-site decimation (a) The Green's function amplitude ϕ_j, ψ_j due to a source applied to the left nodal site of phenyl ring l. (b) An equivalent lattice with two edges.

For a finite macrocycle of N phenyl rings, and for a source connected to the left nodal site of phenyl ring l, one again finds $\phi_j = 0$ for all j, while the Green's function amplitudes on the right nodal sites of the phenyl rings are

$$\psi_j = \frac{1}{2}\left(\frac{1}{2}\right)^{(j-l)}[\Theta(j-l) + a] \tag{31}$$

In this expression

$$a = \frac{1}{(2^N - 1)} \tag{32}$$

and $\Theta(j-l)$ is a step function defined by $\Theta(j-l) = 1$ for $j \geq l$ and $\Theta(j-l) = 0$ for $j < l$. Note that ψ_j satisfies the periodic boundary condition $\psi_0 = \psi_N$. Equation (31) shows that in contrast with a doubly infinite linear chain (described by equation 30) ψ_j does not vanish when

$j < l$. Instead it is proportional to the constant a and grows exponentially with decreasing j. Similarly, for a source acting on the right nodal site of phenyl ring l, whose Green's function amplitude is denoted ψ_l, one finds $\psi_j = 0$ for all j and

$$\phi_j = \frac{1}{2}\left(\frac{1}{2}\right)^{(l-j)}[\Theta(l-j) + a] \tag{33}$$

The modulus squared of these functions reproduce precisely the behaviour of the tight-binding results in Figure 4 and are analytic formulae for the corresponding magic number table Figure S4.

S8. Edge states of diatomic chains and rings based on TBMs.

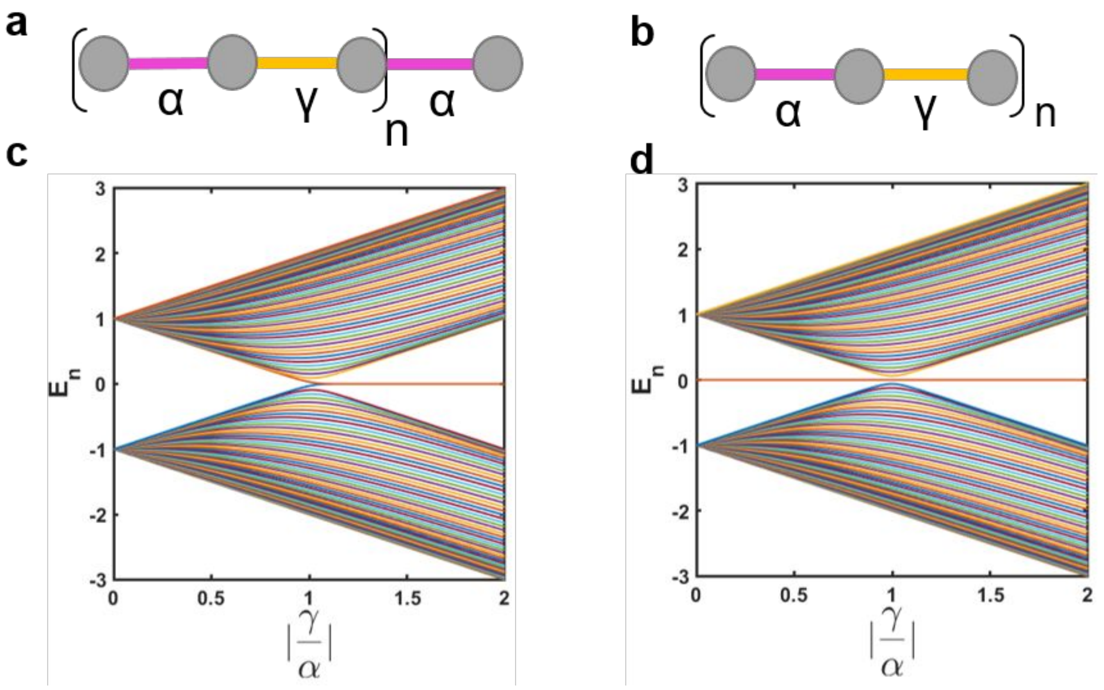

Figure S16. (a) A TBM for a diatomic chain terminated with the same bond. (b) A TBM for a diatomic chain terminated with the different bonds. (c) Eigenvalues versus the ratio of the two

bonds $\left|\frac{\gamma}{\alpha}\right|$ for model in a. (d) Eigenvalues versus the ratio of the two bonds $\left|\frac{\gamma}{\alpha}\right|$ for model in b. In both cases, the site energies are chosen to be zero, $\alpha = 1$ and γ is varied from 0 to 2 in the two models.

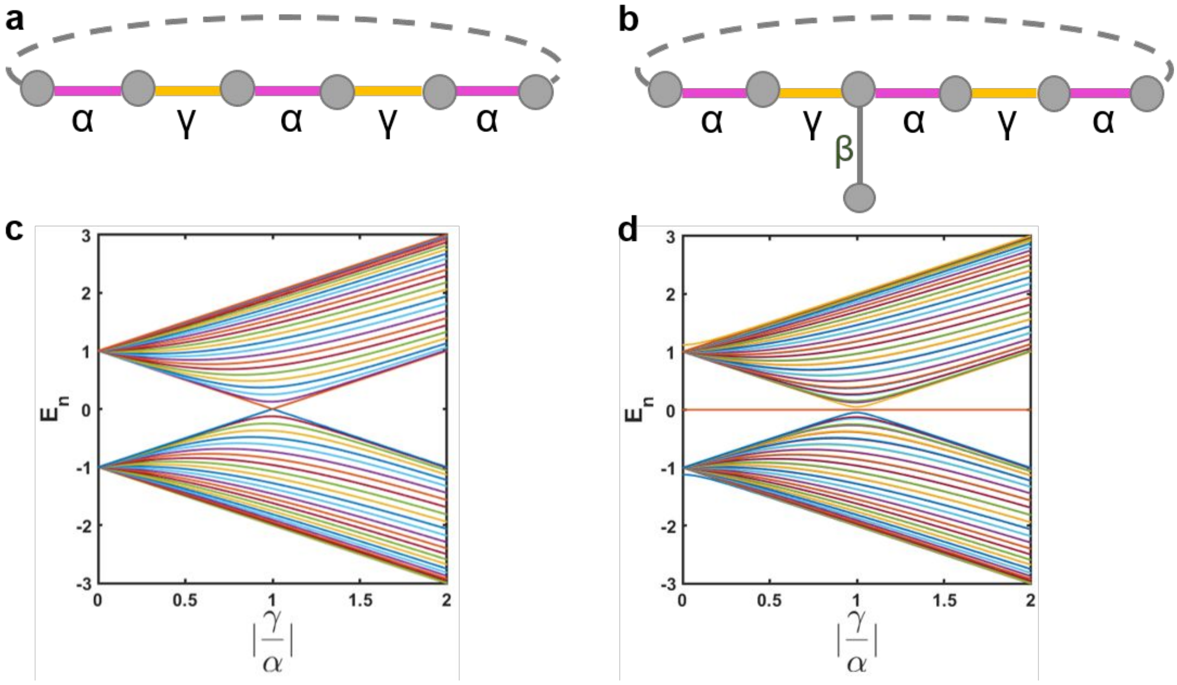

Figure S17 (a) TBM for a diatomic macrocycle. (b) TBM for a diatomic macrocycle with a pendent site. (c) Eigenvalues versus the ratio $\left|\frac{\gamma}{\alpha}\right|$ for model in a. (d) Eigenvalues versus the ratio $\left|\frac{\gamma}{\alpha}\right|$ for model in b. In both cases, the site energies are chosen to be zero, $\alpha = 1$ and γ is varied from 0 to 2 in the two models.

S9. METHODS

1. Connectivity theory in more detail

In its simplest form[3,4–6], the theory starts by constructing a connectivity table C of each molecule, with entries C_{ij} in row i and column j. If sites i and j are nearest neighbours, then

C_{ij} is assigned the number 1. All other entries are set to $zero$. Figure S3 shows the connectivity table for CPP6. For such a bipartite lattice, where odd-numbered sites connect to even-numbered sites only, C is block off-diagonal, of the form

$$C = \begin{pmatrix} 0 & \overline{C}^t \\ \overline{C} & 0 \end{pmatrix} \tag{34}$$

(where $\overline{C}^t$ is the transpose of $\overline{C}$)and therefore only the sub-matrix $\overline{C}^t$ is shown in Figure S3. For comparison, the connectivity table for the linear chain of 6 rings is obtained by setting the coupling between the left-most and right-most sites (6 and 33) to $zero$. (see entry highlighted in magenta in Figure S3). These entries reflect the fact that sites 6 and 33 of the macrocycle are nearest neighbours, due to the periodic boundary conditions. In contrast, sites 6 and 33 of the linear chain are not nearest neighbours, and therefore for the connectivity table of the linear chain, $C_{6,33} = C_{33,6} = 0$, which reflects the free-end boundary conditions of the chain.

2. Magic number theory

To demonstrate the topological origin of the behaviour in Figure 3b, we now develop a 'magic number theory' of connectivity-dependent transport near $E = 0$. To describe transport near the middle of the HOMO-LUMO gap, we proceed by noting that the Green's function of the isolated molecule is $g(E) = (E - H)^{-1}$, and for a molecule which is weakly coupled to electrodes at sites i,j , the transmission coefficient is proportional to τ_{ij}, where

$$\tau_{ij}(E) = [g_{ij}(E)]^2 \quad (35)$$

$\tau_{ij}(E)$ will be referred to as a 'core transmission coefficient', because in the limit that the macrocycle is weakly coupled to the electrodes and for small values of E, the transmission coefficient $T_{ij}(E)$ of a macrocycle connected to external electrodes is proportional to $\tau_{ij}(E)$. Since $g(0) = -H^{-1}$ and $H = -C$, mid-gap transport is controlled by a 'magic number table' denoted M and defined by

$$M = dC^{-1}, \quad (36)$$

where d is any constant, chosen for convenience. The inverse C^{-1} could be computed using one line of MATLAB (ie $= d * \mathrm{inv}(C\ )$) or alternatively as shown below, to reveal generic trends, it can be calculated analytically. As shown in appendix F, for $\frac{E}{\Delta} \ll 1$, $g(E)$ can be approximated by

$$g(E) \approx M + EM^2 \quad (37)$$

The magic number tables for the 6-ring macrocycle and chain are shown in Figures S4 and S10. For a bipartite lattice, since C is block off-diagonal, M is also block off-diagonal and of the form

$$M = \begin{pmatrix} 0 & \overline{M}^t \\ \overline{M} & 0 \end{pmatrix}, \quad (38)$$

$$\tau_{ij}(E) \approx [M_{ij}]^2 \quad \text{(CQI)} \quad (39)$$

whereas if i is odd and j is even, or vice versa, corresponding to CQI connectivities (see Figure S4), the core transmission function is proportional to E^2 and of the form

$$\tau_{ij}(E) \approx E^2[D_{ij}]^2 \qquad \text{(DQI)} \qquad (40)$$

Clearly in latter (DQI) case, $\tau_{ij}(E)$ vanishes at the gap centre, corresponding to $E = 0$. In these equations, the magic number tables M and D are given by

$$\overline{M} = [\overline{C}^t]^{-1} \quad \text{and} \quad \overline{D} = \overline{M}\overline{M}^t \qquad (41)$$

As discussed in appendix H of the Supporting Information, the simplest tight-binding Hamiltonian H describing such a molecule is obtained from C via the relation $H = -C$ and the eigenvalues of H yield the molecular orbital (MO) energies. Since the lattice is bipartite, to each positive MO energy, there is a corresponding negative MO energy. If the energy of the highest occupied molecular orbital (HOMO) and lowest unoccupied molecular orbital (LUMO) are E_H and E_L respectively, then the HOMO-LUMO gap is $\Delta = E_L - E_H$ and the middle of the HOMO-LUMO gap is $\frac{E_H + E_L}{2}$. Since the lattice of sites is bipartite, $E_L = -E_H$ and $\frac{E_H + E_L}{2} = 0$. In a single-molecule junction, electrons are injected into the molecule with energies E close to the Fermi energy of the external electrodes. Typically this lies close to the middle of the HOMO-LUMO gap, such that $\frac{E}{\Delta} \ll 1$. Therefore it is of interest to develop a mid-gap theory of electron transport for values of E close to zero (see Figure S5).

3. DFT calculations

SIESTA[7]employs norm-conserving pseudopotentials to account for the core electrons and linear combinations of atomic orbitals to construct the valence states. The generalized gradient approximation (GGA) of the exchange and correlation functional is used with the Perdew−Burke−Ernzerhof parametrization (PBE)[8] a double-ζ polarized (DZP) basis set, a real-space grid defined with an equivalent energy cutoff of 200 Ry. The geometry optimization for each structure is performed to the forces smaller than 10 meV/Å.

4. Transport theory

The transmission coefficient $T(E)$ for electrons of energy E (passing from the source to the drain) can be calculated by the following relation:

$$T(E) = Trace\left(\Gamma_R(E)G^R(E)\Gamma_L(E)G^{R\dagger}(E)\right) \qquad (42)$$

the expression, $\Gamma_{L,R}(E) = i\left(\Sigma_{L,R}(E) - \Sigma_{L,R}{}^{\dagger}(E)\right)$ describes the level broadening due to the coupling between left (L) and right (R) electrodes and the central scattering region, $\Sigma_{L,R}(E)$ are the retarded self-energies associated with this coupling and $G^R(E) = (ES - H - \Sigma_L - \Sigma_R)^{-1}$ is the retarded Green's function, where H is the Hamiltonian and S is overlap matrix. Using obtained transmission coefficient $T(E)$, the electrical conductance G could be calculated by Landauer formula $(G = G_0 \int dE\, T(E)(-\partial f(E)/\partial E))$, where $G_0 = 2e^2/h$ is conductance quantum, e is the charge of an electron; h is the Planck's constant; $f(E) = (1 + exp$

$\left((E - E_F)/k_B T\right)^{-1}$ is the Fermi−Dirac distribution function; E_F is the Fermi energy; T is the temperature, and $k_B = 8.6 \times 10^{-5}\ eV/K$ is Boltzmann's constant.

5. Tight-Binding Model of the π_z systems.

This description represents each molecule by a lattice of sites labelled i and located on each carbon atom. These sites represent the π_z orbitals of each carbon, which combine to form the pi system of the molecule. The Hamiltonian of the simple tight-binding model describes a single orbital per atom with nearest-neighbour couplings $\gamma = -1$. All site energies are set to zero, except the site energies of nitrogen is $\varepsilon_n = -0.5$.

Appendices

In the remaining part of this Supporting Information, we present a series of appendices containing analytic results for relevant Green's functions and edge states. For completeness and to establish a common notation, we also include some notes about topological insulators, chiral symmetries and connectivity theory.

Appendix A. Green's function due to a source at site $(l)_q$.

The Green's function G due to a source acting on the site $(l)_q$ has matrix elements $\begin{pmatrix} G_{(j)_p(l)_q} \\ G_{(j)_q(l)_q} \end{pmatrix}$ $= \begin{pmatrix} \phi_j \\ \psi_j \end{pmatrix}$ and instead of equation (1), it satisfies

$$\begin{pmatrix} \epsilon\phi_j + \alpha\psi_j \\ \epsilon\psi_j + \alpha\phi_j \end{pmatrix} = -\gamma \begin{pmatrix} \psi_{j-1} \\ \phi_{j+1} \end{pmatrix} + \begin{pmatrix} 0 \\ 1 \end{pmatrix}\delta_{jl} \qquad \text{(A.1)}$$

For $\epsilon = 0$, taking into account the two possible solutions (11), the Green's function due to a source at site $(l)_q$, the Green's function must therefore have the form

$$\begin{pmatrix} \phi_j \\ \psi_j \end{pmatrix} = \frac{1}{\alpha}\left[\begin{pmatrix} 1 \\ 0 \end{pmatrix} e^{-\beta(j-l)} e^{i\pi s(j-l)} \Theta(l-j) + a\begin{pmatrix} 0 \\ 1 \end{pmatrix} e^{\beta(j-l)} e^{i\pi s(j-l)} + b\begin{pmatrix} 1 \\ 0 \end{pmatrix} e^{-\beta(j-l)} e^{i\pi s(j-l)}\right] \text{(A.2)}$$

For $j = l$, equation (1) yields (since $\epsilon = 0$),

$$\gamma\psi_{l-1} + \alpha\psi_l = 0 \qquad \text{and} \qquad \gamma\phi_{l+1} + \alpha\phi_l = 1 \qquad \text{(A.3)}$$

Substituting the expression (A.2) into these shows that equations (A.3) are satisfied for any choice a and b. On the other hand, from figure S13, periodic boundary conditions require

$$\phi_1 = \phi_{N+1} \qquad \text{and} \qquad \psi_0 = \psi_N \qquad \text{(A.4)}$$

Substituting the expression (A.2) into these shows that the second of equations (A.4) can only be satisfied if $a = 0$ and therefore

$$\begin{pmatrix} G_{(j)_p(l)_q} \\ G_{(j)_q(l)_q} \end{pmatrix} = \frac{1}{\alpha}\begin{pmatrix} 1 \\ 0 \end{pmatrix} e^{-\beta(j-l)} e^{i\pi s(j-l)} [\Theta(l-j) + b] \qquad \text{(periodic chain) (A.5)}$$

Substituting the expression (A.5) into first of equations (A.4) yields

$$e^{-\beta(1-l)} e^{i\pi s(1-l)} [1 + b] = b e^{-\beta(N+1-l)} e^{i\pi s(N+1-l)}$$

Ie
$$b = \frac{e^{\beta N} e^{i\pi s N}}{(1 - e^{\beta N} e^{i\pi s N})} \qquad \text{(A.6)}$$

For a chain with free ends, the boundary conditions (A.4) are replaced by

$$\phi_{N+1} = 0 \qquad \text{and} \qquad \psi_0 = 0 \tag{A.7}$$

Substituting equation (A.2) into these yields $a = b = 0$ and hence the Green's function of a linear chain is

$$\begin{pmatrix} G_{(j)_p(l)_q} \\ G_{(j)_q(l)_q} \end{pmatrix} = \frac{1}{\alpha}\begin{pmatrix} 1 \\ 0 \end{pmatrix} e^{-\beta(j-l)} e^{i\pi s(j-l)} \Theta(l-j) \qquad \text{(linear chain)} \qquad \text{(A.8)}$$

Appendix B. Expressions for the full Green's function *G*.

So far, we have computed Green's function matrix elements between nodal sites only. For the structure shown in Figure S13, we now compute the Green's function for all sites. For macrocycle of N cells with periodic boundary conditions or a linear chain of N cells, sites are labelled $(i)_r$, where $i = 1,2,\ldots N$ and for cells composed of m sites, $r = 1,2\ldots m$. For the structure of Figure S12, $m = 6$. Sites which connect to neighbouring cells will be referred to as 'nodal sites,' while sites which do not connect to neighbouring cells are 'non-nodal sites.' In what follows, arbitrary sites such as r or s belonging to cell j will be labelled $(j)_r$ or $(j)_s$. For the structure of figure S12, the two nodal sites of cell j are denoted $(j)_1$ and $(j)_4$. More generally, the fixed symbols p and q are used to denote nodal sites only, and the two nodal sites of cell j are denoted $(j)_p$ and $(j)_q$.

In what follows, it is convenient to write $N_1 = 2N$ and $N_0 = mN$. The full Green's function G and the is a $N_0 x N_0$ matrix, which can be written in the form

$G = \begin{pmatrix} G_{11} & G_{10} \\ G_{01} & G_{00} \end{pmatrix}$, where G_{11} is a $N_1 x N_1$ matrix containing matrix elements of the full Green's function between nodal sites only, G_{00} is a $(N_0 - N_1)x(N_0 - N_1)$ matrix containing matrix elements of the full Green's function between non-nodal sites only and G_{10} is a rectangular matrix containing elements between nodal and non-nodal sites.

Then if $\gamma = 0$, then the $N_1 x N_1$ matrix G_{11} reduces to the Green's function between all nodal sites $(j)_p$ and $(l)_q$ of a set of N disconnected cells, denoted g_{11}. For example, from equation (20), if the cells are phenyl ring, the matrix elements of g_{11} at $E = 0$ are

$$g_{(j)_1(l)_1} = g_{(j)_4(l)_4} = 0 \text{ and } g_{(j)_1(l)_4} = g_{(j)_4(l)_1} = -\frac{1}{2\gamma}\delta_{jl}. \qquad \text{(B.1)}$$

These are the sub-blocks of a $2Nx2N$ matrix denoted g_{11}.

More broadly, when $\gamma = 0$, the $N_0 x N_0$ matrix G reduces to the $N_0 x N_0$ matrix g, with non-zero matrix elements between sites belonging to the same cell only. For example, from equation (18), if the cells are phenyl ring, the matrix elements of g are given by equation (18); ie

$$g_{(j)_r(l)_s} = \hat{g}_{rs}\delta_{il} \qquad \text{(B.2)}$$

where $\hat{g}_{rs}$ is is given by equation (18). If the $6x6$ Hamiltonian of an isolated phenyl ring is h, then equation (18) is equivalent to writing $\hat{g} = (E - h)^{-1}$.

When $\gamma = 0$, the full Hamiltonian H reduces to a $N_0\, xN_0$ matrix H_0, with matrix elements

$$(H_0)_{(j)_r(l)_q} = h_{pq}\delta_{ij} \qquad \text{(B.3)}$$

When $\gamma \neq 0$, the Hamiltonian is a $N_0 x N_0$ matrix $H = H_0 + H_1$, where the $N_0 x N_0$ matrix H_1 has a $N_1 x N_1$ non-zero sub-matrix denoted h_{11} containing the couplings between nodal sites of phenyl rings. Ie H_1 has the structure

$$H_1 = \begin{pmatrix} h_{11} & 0 \\ 0 & 0 \end{pmatrix} \qquad \text{(B.4)}$$

For the structure of figure S18, the only non-zero matrix elements of h_{11} are

$$(h_{11})_{(j)_4(j')_1} = (h_{11})_{(j')_1(j)_4} = -\gamma \qquad \text{(B.5)}$$

where in the presence of periodic boundary conditions, $j' = j + 1 \; mod \; N$.

Starting from the disconnected system described by the Hamiltonian H_0 of dimensionality $N_0 = mN$, with a known Green's function g satisfying $(E - H_0)g = I$, our task is to compute the new Green's function G of the full Hamiltonian is $H = H_0 + H_1$.

Conceptually the introduction of h_{11} leads to the partitioning of the whole structure into $N_1 x$ N_1 and $(N_0 - N_1)x(N_0 - N_1)$ substructures denoted 1 and 0 respectively and coloured yellow and blue in figure S18. Therefore it is convenient to express both matrices G and g in terms of submatrices as follows:

$$G = \begin{pmatrix} G_{11} & G_{10} \\ G_{01} & G_{00} \end{pmatrix} \quad \text{and} \quad g = \begin{pmatrix} g_{11} & g_{10} \\ g_{01} & g_{00} \end{pmatrix} \tag{B.6}$$

The sub-blocks of a $2Nx2N$ matrix denoted G_{11} are given by equation (25). Our task is to compute the other sub-blocks of G.

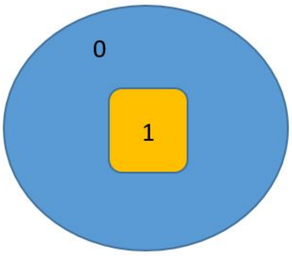

Figure S18. A quantum structure ‘0’ containing a finite region ‘1’ (shaded yellow), which is subject to a local perturbation.

As described in chapter 11 of ref. [3], to obtain the remaining sub-blocks, we need to solve the equation

$$(EI - H_0 - H_1)G = I. \tag{B.7}$$

Since all matrices are finite, equation (32) is equivalent to

$$G(EI - H_0 - H_1) = I. \tag{B.8}$$

After defining $g = (EI - H_0)^{-1}$, the solutions to these are

$$G = g + gH_1G \text{ and } \qquad G = g + GH_1g \tag{B.9}$$

Substituting the second solution into the first yields

$$G = g + gH_1g + gH_1GH_1g \tag{B.10}$$

Selecting sub-blocks involving the subspace 0 yields

$$G_{10} = g_{10} + G_{11}h_{11}g_{10} \tag{B.11}$$

$$G_{01} = g_{01} + g_{01}h_{11}G_{11} \tag{B.12}$$

$$G_{00} = g_{00} + g_{01}h_{11}g_{10} + g_{01}h_{11}G_{11}h_{11}g_{10} \tag{B.13}$$

Since all matrices on the right hand side are known, this completes the solution.

Since odd-odd and even-even mid-gap Green’s functions vanish, after noting the Kronecka delta in equation (27), equation (B.11) yields

$$G_{(j)_1(l)_2} = g_{(j)_1(l)_2} + G_{(j)_1(l-1)_4}(-\gamma)g_{(l)_1(l)_2}$$

Ie $$G_{(j)_1(l)_2} = \hat{g}_{12}\delta_{jl} + G_{(j)_1(l-1)_4}(-\gamma)\hat{g}_{12} \quad \text{(B.14)}$$

Similarly, equation (B.13) yields

$$G_{(j)_2(l)_3} = g_{(j)_2(l)_3} + g_{(j)_2(j)_1}(-\gamma)g_{(j-1)_4(l)_3} + g_{(j)_2(j)_1}(-\gamma)G_{(j-1)_4(l+1)_1}(-\gamma)g_{(l)_4(l)_3}$$

Ie $$G_{(j)_2(l)_3} = \hat{g}_{23}\delta_{jl} + \hat{g}_{21}(-\gamma)\hat{g}_{43}\delta_{j-1,l} + \hat{g}_{21}(-\gamma)G_{(j-1)_4(l+1)_1}(-\gamma)\hat{g}_{43}$$
(B.15)

Also, equation (B.12) yields

$$G_{(j)_3(l)_4} = g_{(j)_3(l)_4} + g_{(j)_3(j)_4}(-\gamma)G_{(j+1)_1(l)_4}$$

Ie $$G_{(j)_3(l)_4} = \hat{g}_{34}\delta_{jl} + \hat{g}_{34}(-\gamma)G_{(j+1)_1(l)_4} \quad \text{(B.16)}$$

Note that by symmetry $G_{(l)_2(j)_1} = G_{(j)_1(l)_2}$ and $G_{(j)_2(l)_3} = G_{(l)_3(j)_2}$. Also note that from equation (19) $\hat{g}_{12} = \hat{g}_{21} = \hat{g}_{23} = \hat{g}_{43} = \frac{1}{2\gamma'}$. Hence, for the case $\gamma' = \gamma$,

$$G_{(j)_1(l)_2} = \frac{1}{2\gamma}\delta_{jl} - \frac{1}{2}G_{(j)_1(l-1)_4} \quad \text{(B.17)}$$

$$G_{(j)_2(l)_3} = \frac{1}{2\gamma}[\delta_{jl} - \frac{1}{2}\delta_{j-1,l}] + \frac{1}{4}G_{(j-1)_4(l+1)_1} \quad \text{(B.18)}$$

$$G_{(j)_3(l)_4} = \frac{1}{2\gamma}\delta_{jl} - \frac{1}{2}G_{(j+1)_1(l)_4} \quad \text{(B.19)}$$

Examples for a periodic chain.

For a periodic chain, these yield

$$G_{(j)_3(l)_4} = \frac{1}{2\gamma}[\delta_{jl} + \frac{1}{2}2^{(j+1-l)}a] \quad \text{(B.20)}$$

Therefore $$G_{(j)_3(j)_4} = \frac{1}{2\gamma}[1 + a] \quad \text{(B.21)}$$

Also $$G_{(j)_2(l)_3} = G_{(l)_3(j)_2} = \frac{1}{2\gamma}[\delta_{jl} - \frac{1}{2}\delta_{j-1,l}] + (\frac{-1}{2\gamma})2^{-(j-l)}[\Theta(j-l-2) + a] \quad \text{(B.22)}$$

Therefore $$G_{(j)_2(j)_3} = G_{(j)_3(j)_2} = \frac{1}{2\gamma}[1 - a] \quad \text{(B.23)}$$

Hence the ratio $\frac{G_{(j)_3(j)_2}}{G_{(j)_3(j)_4}} = \frac{1-a}{1+a}$, where $a = \frac{2^{-N}}{(1-2^{-N})} = \frac{1}{(2^N-1)}$

Ie $$\frac{G_{(j)_3(j)_2}}{G_{(j)_3(j)_4}} = 1 - \frac{1}{2^{N-1}} \quad \text{(B.24)}$$

Similarly

$$G_{(j)_1(l)_2} = \frac{1}{2\gamma}\delta_{jl} + \frac{1}{2\gamma}2^{(j-l)}[\Theta(l-j-1) + a] \quad \text{(B.25)}$$

Therefore $$G_{(j)_1(j)_2} = \frac{1}{2\gamma}[1 + a] \tag{B.26}$$

As expected, this is equal to $G_{(j)_3(j)_4}$.

The presence of the factor $\frac{1}{2^{N-1}}$ in equation (B.24) explains why magic numbers between non-nodal sites in the table of figure S4 are not necessarily integers.

Examples for a linear chain.

For a linear chain, equation (B.17) to (B.19) yield

$$G_{(j)_3(l)_4} = \frac{1}{2\gamma}[\delta_{jl} + 2^{(j-l)}\Theta(l - 1 - j)] \tag{B.27}$$

Therefore $$G_{(j)_3(j)_4} = \frac{1}{2\gamma} \tag{B.28}$$

Also $$G_{(j)_2(l)_3} = G_{(l)_3(j)_2} = \frac{1}{2\gamma}[\delta_{jl} - \frac{1}{2}\delta_{j-1,l}] + (\frac{-1}{2\gamma})2^{-(j-l)}\Theta(j - l - 2) \tag{B.29}$$

Therefore $$G_{(j)_2(j)_3} = G_{(j)_3(j)_2} = \frac{1}{2\gamma} \tag{B.30}$$

Hence $$\frac{G_{(j)_3(j)_2}}{G_{(j)_3(j)_4}} = 1 \tag{B.31}$$

Similarly

$$G_{(j)_1(l)_2} = \frac{1}{2\gamma}\delta_{jl} + \frac{1}{2\gamma}2^{(j-l)}\Theta(l - 1 - j) \tag{B.32}$$

Therefore $$G_{(j)_1(j)_2} = \frac{1}{2\gamma} \qquad \text{(B.32)}$$

Note that this is equal to $G_{(j)_3(j)_4}$.

Appendix C. Topological insulators.

As a reminder of well-known properties of edge states, we note that from equation (11), a general eigenstate at $E = 0$ is

$$\begin{pmatrix}\phi'_j\\ \psi'_j\end{pmatrix} = A\begin{pmatrix}1\\0\end{pmatrix}e^{-\beta j}e^{i\pi sj} + B\begin{pmatrix}0\\1\end{pmatrix}e^{\beta j}e^{i\pi sj} \tag{C.1}$$

For a semi-infinite lead, with $= 1, ...\infty$, we require

$$\psi_0 = 0 \tag{C.2}$$

Therefore $B = 0$ and provided $\beta > 0$ there is allowed edge state on the left, which decays at ∞.

Writing $A = A'e^{\beta}e^{i\pi s}$ yields

$$\begin{pmatrix}\phi_j^L \\ \psi_j^L\end{pmatrix} = A'\begin{pmatrix}1 \\ 0\end{pmatrix}e^{-\beta(j-1)}e^{i\pi s(j-1)} \tag{C.3}$$

where A' is the amplitude of the state on cell j.

Similarly for a semi-infinite lead, with $= N, N-1, ... -\infty$, we require

$$\phi_{N+1} = 0 \tag{C.4}$$

Therefore $A = 0$ and provided $\beta > 0$ there is allowed edge state on the right, which decays at $-\infty$. Writing $B = B'e^{-\beta N}e^{-i\pi sN}$ yields for this edge state

$$\begin{pmatrix}\phi_j^R \\ \psi_j^R\end{pmatrix} = B'\begin{pmatrix}0 \\ 1\end{pmatrix}e^{\beta(j-N)}e^{i\pi s(j-N)} \tag{C.5}$$

From equation (8), the condition $\beta > 0$ means that

$$\left|\frac{\gamma}{\alpha}\right| > 1 \tag{C.6}$$

In both cases, the semi-infinite lead starts with the bond -α. Hence we conclude that an edge state appears when a semi-infinite lead starts with the weaker bond and does not appear when the lead starts with the stronger bond.In the two-dimensional parameter space of α and γ, this is called the topological region of parameter space and the above edge states are called topological states. When $\left|\frac{\gamma}{\alpha}\right| < 0$, these $E = 0$ states do not exist, because they would diverge at $\pm\infty$.

The case of a finite chain of *N* cells.

For a finite chain of length N, both of the boundary conditions in equations (16) must be satisfied, which yields $A' = B' = 0$ and therefore there are no $E = 0$ edge states. The edge states do not simply disappear. They move to energies $E = \pm E_1$, where $E_1 \to 0$ as $N \to \infty$.

Note that for $\beta > 0$

$$\sum_{j=1}^{\infty} |\phi_j^L|^2 = |A'|^2 \sum_{j=1}^{\infty} e^{-2\beta(j-1)} = \frac{|A'|^2}{1 - e^{-2\beta}}$$

Therefore choosing $|A'|^2 = |B'|^2 = 1 - e^{-2\beta}$ would yield normalised states. Hence it is convenient to write

$$\begin{pmatrix} \phi_j^L \\ \psi_j^L \end{pmatrix} = a_L \begin{pmatrix} c \\ 0 \end{pmatrix} e^{-\beta(j-1)} e^{i\pi s(j-1)} \qquad \text{(C.6)}$$

$$\begin{pmatrix} \phi_j^R \\ \psi_j^R \end{pmatrix} = a_R \begin{pmatrix} 0 \\ c \end{pmatrix} e^{\beta(j-N)} e^{i\pi s(jN)} \qquad \text{(C.7)}$$

where $c^2 = 1 - e^{-2\beta}$.

Appendix D. Chiral symmetry.

As a reminder of well-known properties of bipartite lattices, a Hamiltonian is said to possess chiral symmetry if an operator Γ can be found such that

$\Gamma^\dagger = \Gamma$, $\Gamma^2 = 1$ and $\Gamma H \Gamma^\dagger = -H$. Eg if H is bipartite, such that

$H = \begin{pmatrix} 0 & h \\ \mathrm{h}^\dagger & 0 \end{pmatrix}$ and $\Gamma = \begin{pmatrix} I & 0 \\ 0 & -I \end{pmatrix}$, then $H\Gamma^\dagger = \begin{pmatrix} 0 & h \\ \mathrm{h}^\dagger & 0 \end{pmatrix}\begin{pmatrix} I & 0 \\ 0 & -I \end{pmatrix} = \begin{pmatrix} 0 & -h \\ \mathrm{h}^\dagger & 0 \end{pmatrix}$ and

$$\Gamma H \Gamma^\dagger = \begin{pmatrix} I & 0 \\ 0 & -I \end{pmatrix}\begin{pmatrix} 0 & -h \\ \mathrm{h}^\dagger & 0 \end{pmatrix} = \begin{pmatrix} 0 & -h \\ -\mathrm{h}^\dagger & 0 \end{pmatrix} = -H$$

This means that $H\Gamma^\dagger = -\Gamma H$

If $H|\psi\rangle = E|\psi\rangle$, then consider the state $|\phi\rangle = \Gamma^\dagger|\psi\rangle$. This satisfies

$H|\phi\rangle = \mathrm{H}\Gamma^{\dagger}|\psi\rangle = -\Gamma H|\psi\rangle = -E\Gamma|\psi\rangle = -E|\phi\rangle$. Hence to each eigenstate $|\psi\rangle$ with eigenvalues E, there is another eigenstate $|\phi\rangle$ with eigenvalue $-E$. Ie the eigenvalue spectrum is symmetric about $E = 0$.

Appendix E. Edge states and Green's functions.

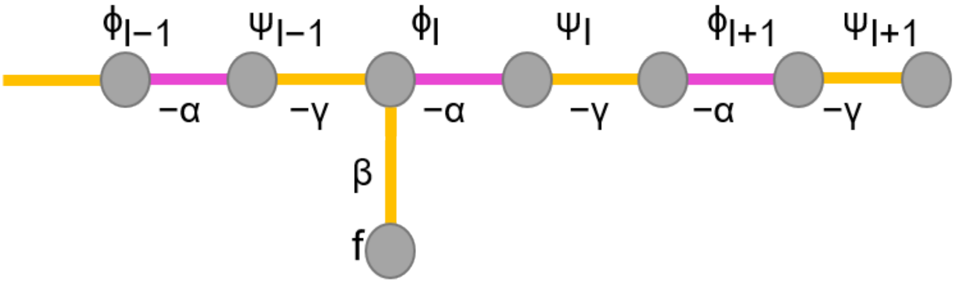

By analogy with equation (1), for $\epsilon = 0$, writing $X = \beta f$, one obtains the following Schrödinger equation

$$\alpha\begin{pmatrix}\psi_j\\ \phi_j\end{pmatrix} = -\gamma\begin{pmatrix}\psi_{j-1}\\ \phi_{j+1}\end{pmatrix} + \begin{pmatrix}X\\ 0\end{pmatrix}\delta_{jl} \qquad \text{(a)}$$

Hence

$$\frac{\phi_{j+1}}{\phi_j} = -\alpha/\gamma \qquad \text{for all } j \qquad \text{(b)}$$

$$\frac{\psi_j}{\psi_{j-1}} = -\gamma/\alpha \qquad \text{for } j > l \qquad \text{(c)}$$

$$\alpha\psi_l + \gamma\psi'_{l-1} = X \qquad \text{(d)}$$

$$\frac{\psi'_{j-1}}{\psi'_j} = -\alpha/\gamma \qquad \text{for } j < l \qquad \text{(e)}$$

(Note that equation (e) is simply a rearrangement of equation (c)). For a double infinite chain equation (b) implies $\phi_j = 0$ for all j, otherwise $|\phi_j| \to \infty$ at either $j \to \infty$ or $j \to -\infty$. In equation (e), since the functional form of the wave function on the left (ie for $j < l$) may be different from that on the right, the wave function on the left is denoted ψ'_j for $j < l$.

Consider the case $\left|\frac{\gamma}{\alpha}\right| > 1$.

In this case, equation (c) implies that $|\psi_j| \to \infty$ as $j \to \infty$. Hence if $\left|\frac{\gamma}{\alpha}\right| > 1$, $\psi_j = 0$ for $j > l$. From equation (c), $\psi_{j-1} = -\frac{\alpha}{\gamma}\psi_j$, so since $\psi_{l+1} = 0$, one finds $\psi_l = 0$. From equation (d), this yields $\psi'_{l-1} = X/\gamma$, so $\psi'_{l-1} \neq 0$, provided $X \neq 0$. Hence from equation (e), since $|\alpha/\gamma| < 1$, ψ'_j decays to zero for $j \to -\infty$. Hence an edge state appears on the left

Consider the case $\left|\frac{\gamma}{\alpha}\right| < 1$.

In this case, equation (c) implies that $|\psi'_j| \to \infty$ as $j \to -\infty$. Hence if $\left|\frac{\gamma}{\alpha}\right| < 1$, $\psi'_j = 0$ for $j < l$. From equation (d), this yields $\psi_l = X/\alpha$, so $\psi_l \neq 0$, provided $X \neq 0$. Hence from equation (c), since $|\gamma/\alpha| < 1$, ψ_j decays to zero for $j \to +\infty$. Hence an edge state appears on the right. In summary, if the bond to the right of ϕ_l has a great magnitude than the bond to the left, then and edge state appears on the right, and vice versa. Since all $\phi_j = 0$, one can view the above structure as follows:

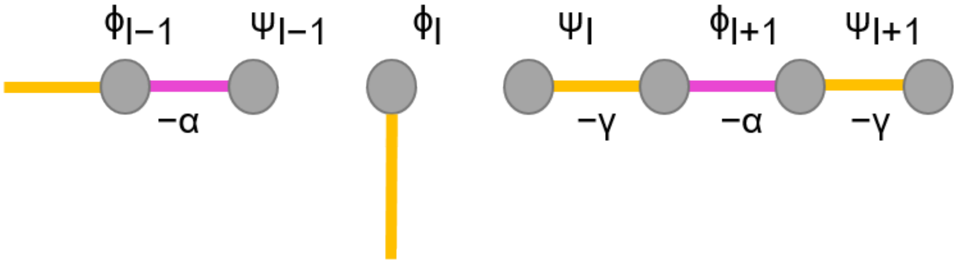

This consists of two semi-infinite leads. The one on the right starts with a bond $-\gamma$, while the semi-infinite lead on the left starts with a bond $-\alpha$. As noted in appendix C, for a semi-infinite lead starting with the weaker of the two bonds, an edge state exists, so an edge state appears in the right lead if $|\gamma| < |\alpha|$ and no state appears in the left lead. Similarly an edge state appears in the left lead if $|\gamma| > |\alpha|$ and no state appears in the right lead. In summary, the Green's function is an edge state.

Appendix F. Green's functions at small, but finite energy *E*.

For a bipartite lattice, the middle of the HOMO-LUMO gap is located at $E = 0$. For a non-bipartite lattice, the middle of the HOMO-LUMO gap does not coincide with $E = 0$, but may lie in the vicinity of $E = 0$. Therefore it is of interest to evaluate $g(E)$ for small, but finite E. To this end, note that

$$g = (E-H)^{-1} = -\left[H(1-EH^{-1})\right]^{-1} = -\left[(1-EH^{-1})\right]^{-1}H^{-1} = -\left[1 + EH^{-1} + \frac{E^2H^{-2}}{2} + \ldots\right] H^{-1}$$

Ie

$$g = -\left[H^{-1} + EH^{-2} + E^2H^{-3} + \ldots\right]$$

Define the matrix $m_n = H^{-n}$, with matrix elements $(m_n)_{ij}$. Then

$$g_{ij} = -\left[(m_1)_{ij} + E(m_2)_{ij} + \frac{E^2(m_3)_{ij}}{2} + \ldots\right]$$

In the notation of the main text, this is written

$$g_{ij} = \left[M_{ij} + ED_{ij} + \frac{E^2(m_3)_{ij}}{2} + \ldots\right]$$

Where $M = -H^{-1}$ and $D = -H^{-2} = -M^2$

Define the pseudo-transmission coefficient $\tau_{ij} = (g_{ij})^2$. Then to order E^2,

$$\tau_{ij} \approx A_{ij} + B_{ij}E^2 + C_{ij}E^2 + D_{ij}\,E, \qquad \text{(E.1)}$$

where

$$A_{ij} = [(m_1)_{ij}]^2 \qquad \text{(E.2)}$$

$$B_{ij} = (m_1)_{ij}(m_3)_{ij} \qquad \text{(E.3)}$$

$$C_{ij} = [(m_2)_{ij}]^2 \qquad \text{(E.4)}$$

$$D_{ij} = 2(m_1)_{ij}(m_2)_{ij} = 2\sqrt{A_{ij}B_{ij}} \qquad \text{(E.5)}$$

For a bipartite lattice, $D_{ij} = 0$ and therefore the linear term in E is absent. Hence

$$\tau_{ij} \approx A_{ij} + (B_{ij} + C_{ij})E^2, \qquad \text{(bipartite lattice)} \qquad \text{(E.6)}$$

For a bipartite lattice, for CQI connectivities, $A_{ij} \neq 0$, $B_{ij} \neq 0$ and $C_{ij} = 0$, and therefore

$$\tau_{ij} \approx A_{ij} + B_{ij}E^2 \qquad \text{(CQI connectivitites of a bipartite lattice) (E.7)}$$

For small E, the leading contribution to τ_{ij} is given by

$$\tau_{ij} \approx A_{ij} \qquad \text{(CQI connectivitites of a bipartite lattice)} \qquad \text{(E.8)}$$

which is a table of positive numbers, each proportional to the square of a magic number.

In contrast, for DQI connectivities, $A_{ij} = B_{ij} = 0$ and $C_{ij} \neq 0$, and therefore

$$\tau_{ij} \approx C_{ij}E^2 \qquad \text{(DQI connectivitites of a bipartite lattice)} \qquad \text{(E.9)}$$

In summary, equation (E.1) shows that the pseudo-transmission coefficient has a parabolic energy dependence. For a bipartite lattice, the linear term is absent and for DQI connectivities,

τ_{ij} vanishes at the gap centre, with an E^2 energy dependence, given by equation (E.9). Furthermore, since the term $B_{ij}E^2$ is never the leading term, for small E, equation (E.1) can be written

$$\tau_{ij} \approx ((m_1)_{ij} + E(m_2)_{ij})^2, \tag{E.10}$$

which is necessarily positive, as expected. Note that in the notation of the main text, $M = -m_1$ and $D = m_2$

Appendix G. Expressions for m_2 for the chain of Figure S13.

From equation (16) and (17), matrix elements $(m_1)_{(j)_p(l)_p} = (m_1)_{(j)_q(l)_q} = 0$ and $(m_1)_{(j)_q(l)_p} = (m_1)_{(j)_q(l)_p} = G_{(j)_q(l)_p}$, where

$$G_{(j)_q(l)_p} = G_{(l)_p(j)_q} = \frac{1}{\alpha} e^{\beta(j-l)} e^{i\pi s(j-l)} [\Theta(j-l) + a] \tag{G.1}$$

In the above expression,

$$a = 0 \text{ for a linear chain and for a periodic chain, } a = \frac{e^{\beta N} e^{i\pi s N}}{(1 - e^{\beta N} e^{i\pi s N})}. \tag{G.2}$$

For later use, note that for a periodic chain, $e^{2\beta N}(1+a)^2 = \frac{e^{2\beta N}}{(1 - e^{\beta N} e^{i\pi s N})^2}$ and therefore

$$e^{2\beta N}(1+a)^2 = a^2 \tag{G.3}$$

Since $m_2 = m_1^2 = G^2$, one obtains

$$(m_2)_{(i)_p(j)_p} = \sum_{l=1}^{N} G_{(i)_p(l)_q} G_{(l)_q(j)_p}$$

where

$$G_{(i)_p(l)_q}G_{(l)_q(j)_p} = \frac{1}{\alpha^2}e^{\beta(l-i)}e^{i\pi s(i-l)}[\Theta(l-i)+a]\frac{1}{\alpha}e^{\beta(l-j)}e^{i\pi s(l-j)}[\Theta(l-j)+a]$$

Ie

$$G_{(i)_p(l)_q}G_{(l)_q(j)_p} = \frac{1}{\alpha^2}e^{-\beta(i+j)}e^{i\pi s(i+j)}e^{2\beta l}[\Theta(l-i)\Theta(l-j)+a\{\Theta(l-i)+\Theta(l-j)\}+a^2]$$

Define

$$x_j = \sum_{l=j}^{N} e^{2\beta l} = e^{2\beta j}[1+e^{2\beta}+e^{4\beta}+\ldots+\ e^{2\beta(N-j)} = \frac{e^{2\beta j}-e^{2\beta(N+1)}}{1-e^{2\beta}} \qquad \text{(G.4)}$$

Then if k is the greater of i or j,

$$(m_2)_{(i)_p(j)_p} = \frac{1}{\alpha^2}e^{-\beta(i+j)}e^{i\pi s(i+j)}\phi \qquad \text{(G.5)}$$

where

$$\phi = x_k + a(x_i + x_j) + a^2 x_1$$

From equation (G.4),

$$\phi = \frac{e^{2\beta k} + a(e^{2\beta i}+e^{2\beta j}) + a^2 e^{2\beta} - (1+2a+a^2)e^{2\beta(N+1)}}{1-e^{2\beta}}$$

For a linear chain, $a = 0$, so this yields

$$\phi = \frac{e^{2\beta k}-e^{2\beta(N+1)}}{1-e^{2\beta}} \qquad \text{(linear chain)} \qquad \text{(G.6)}$$

For periodic boundary conditions, from equation (G.3), this simplifies to

$$\phi = \frac{e^{2\beta k}+a(e^{2\beta i}+e^{2\beta j})}{1-e^{2\beta}} \qquad \text{(periodic chain(macrocycle))} \qquad \text{(G.7)}$$

Hence equation (G.5) becomes

$$(m_2)_{(i)_p(j)_p} = \frac{e^{i\pi s(i+j)}}{\alpha^2(1-e^{2\beta})}[e^{\beta|i-j|} - e^{\beta(2(N+1)-i-j))}] \quad \text{(linear chain)} \quad \text{(G.8)}$$

$$(m_2)_{(i)_p(j)_p} = \frac{e^{i\pi s(i+j)}}{\alpha^2(1-e^{2\beta})}[e^{\beta|i-j|} + a(e^{\beta|i-j|} + e^{-\beta|i-j|})] \quad \text{(periodic chain(macrocycle))} \qquad \text{(G.8)}$$

The above argument can be repeated to yield $(m_2)_{(i)_q(j)_q}$:

$$(m_2)_{(i)_q(j)_q} = \sum_{l=1}^{N} G_{(i)_q(l)_p}G_{(l)_p(j)_q}$$

where

$$G_{(i)_p(l)_q}G_{(l)_q(j)_p} = \frac{1}{\alpha^2}e^{\beta(l-i)}e^{i\pi s(i-l)}[\Theta(l-i)+a]e^{\beta(l-j)}e^{i\pi s(l-j)}[\Theta(l-j)+a]$$

Ie

$$G_{(i)_p(l)_q}G_{(l)_q(j)_p} = \frac{1}{\alpha^2}e^{-\beta(i+j)}e^{i\pi s(i+j)}e^{2\beta l}[\Theta(l-i)\Theta(l-j)+a\{\Theta(l-i)+\Theta(l-j)\}+a^2]$$

Define

$$x_j = \sum_{l=j}^{N} e^{2\beta l} = e^{2\beta j}[1+e^{2\beta}+e^{4\beta}+\ldots+\ e^{2\beta(N-j)} = \frac{e^{2\beta j} - e^{2\beta(N+1)}}{1-e^{2\beta}} \qquad \text{(G.4)}$$

Then if k is the greater of i or j,

$$(m_2)_{(i)_p(j)_p} = \frac{1}{\alpha^2}e^{-\beta(i+j)}e^{i\pi s(i+j)}\phi \qquad \text{(G.5)}$$

where

$$\phi = x_k + a(x_i + x_j) + a^2 x_1$$

From equation (G.4),

$$\phi = \frac{e^{2\beta k} + a(e^{2\beta i} + e^{2\beta j}) + a^2 e^{2\beta} - (1+2a+a^2)e^{2\beta(N+1)}}{1-e^{2\beta}}$$

For a linear chain, $a = 0$, so this yields

$$\phi = \frac{e^{2\beta k} - e^{2\beta(N+1)}}{1-e^{2\beta}} \qquad \text{(linear chain)} \qquad \text{(G.6)}$$

For periodic boundary conditions, from equation (G.3), this simplifies to

$$\phi = \frac{e^{2\beta k} + a(e^{2\beta i} + e^{2\beta j})}{1-e^{2\beta}} \qquad \text{(periodic chain(macrocycle))} \qquad \text{(G.7)}$$

Hence equation (G.5) becomes

$$(m_2)_{(i)_p(j)_p} = \frac{e^{i\pi s(i+j)}}{\alpha^2(1-e^{2\beta})}[e^{\beta|i-j|} - e^{\beta(2(N+1)-i-j))}] \qquad \text{(linear chain)} \qquad \text{(G.8)}$$

$$(m_2)_{(i)_p(j)_p} = \frac{e^{i\pi s(i+j)}}{\alpha^2(1-e^{2\beta})}[e^{\beta|i-j|} + a(e^{\beta|i-j|} + e^{-\beta|i-j|})] \qquad \text{(periodic chain(macrocycle))} \qquad \text{(G.9)}$$

Similarly

$$(m_2)_{(i)_q(j)_q} = \sum_{l=1}^{N} G_{(i)_q(l)_p} G_{(l)_p(j)_q}$$

Where

$$G_{(i)_q(l)_p} G_{(l)_p(j)_q} = \frac{1}{\alpha^2} e^{\beta(i-l)} e^{i\pi s(i-l)} [\Theta(i-l) + a] e^{\beta(j-l)} e^{i\pi s(j-l)} [\Theta(j-l) + a]$$

Ie

$$G_{(i)_q(l)_p} G_{(l)_p(j)_q} = \frac{1}{\alpha^2} e^{\beta(i+j)} e^{i\pi s(i+j)} e^{-2\beta l} [\Theta(i-l)\Theta(j-l) + a\{\Theta(i-l) + \Theta(j-l)\} + a^2]$$

Define

$$y_j = \sum_{l=1}^{j} e^{-2\beta l} = e^{-2\beta}[1 + e^{-2\beta} + e^{-4\beta} + \ldots + e^{-2\beta(j-1)}] = \frac{e^{-2\beta} - e^{-2\beta(j+1)}}{1 - e^{-2\beta}}$$

Ie $$y_j = \frac{e^{-2\beta j} - 1}{1 - e^{2\beta}} \qquad \text{(G.10)}$$

Then if k is the smaller of i or j,

$$(m_2)_{(i)_p(j)_p} = \frac{1}{\alpha^2} e^{\beta(i+j)} e^{i\pi s(i+j)} \psi \qquad \text{(G.11)}$$

where

$$\psi = y_k + a(y_i + y_j) + a^2 y_N$$

From equation (G.4),

$$\psi = \frac{e^{-2\beta k} + a(e^{-2\beta i} + e^{-2\beta j}) + a^2 e^{-2\beta N} - (1 + 2a + a^2)}{1 - e^{2\beta}}$$

$$e^{2\beta N}(1 + a)^2 = a^2$$

For a linear chain, $a = 0$, so this yields

$$\psi = \frac{e^{-2\beta k} - 1}{1 - e^{2\beta}} \quad \text{(linear chain)} \qquad \text{(G.12)}$$

For periodic boundary conditions, from equation (G.3), this simplifies to

$$\psi = \frac{e^{-2\beta k} + a(e^{-2\beta i} + e^{-2\beta j})}{1 - e^{2\beta}} \quad \text{(periodic chain(macrocycle))} \qquad \text{(G.13)}$$

Hence equation (G.11) becomes

$$(m_2)_{(i)_q(j)_q} = \frac{e^{i\pi s(i+j)}}{\alpha^2(1 - e^{2\beta})}[e^{\beta|i-j|} - e^{\beta(i+j)}] \quad \text{(linear chain)} \quad \text{(G.14)}$$

$$(m_2)_{(i)_q(j)_q} = \frac{e^{i\pi s(i+j)}}{\alpha^2(1-e^{2\beta})}[e^{\beta|i-j|} + a(e^{\beta|i-j|} + e^{-\beta|i-j|})] \text{ (periodic chain(macrocycle))} \quad (G.15)$$

As expected from symmetry, this shows that for a macrocycle $(m_2)_{(i)_p(j)_p} = (m_2)_{(i)_q(j)_q}$, whereas for a linear chain,

$$(m_2)_{(i)_p(j)_p} = (m_2)_{(N+1-i)_q(N+1-j)_q}$$

Appendix H: Connectivity theory.

Connectivity theory describes how the electrical conductance of a molecule varies when the points of contact to external electrodes are changed. In particular, by varying the connectivity of electrodes to a molecule and comparing theory with the resulting changes in electrical or thermal conductance, connectivity theory reveals the underlying quantum interference (QI) effects, which control the flow of electricity of heat through molecules, even at room temperature. A detailed account of the concepts and theoretical tools underlying connectivity theory can be found in the textbook ref [3].

To predict the connectivity dependence of transport properties a molecule, one computes the transmission coefficient $T_{ij}(E)$, which describes how electrons of energy E pass from a source electrode to a drain electrode. If the possible points of contact to the molecule are labelled by integers such as i and j, then $T_{ij}(E)$ is the transmission coefficient associated with connectivity

i,j. Starting from $T_{ij}(E)$, one can then compute the corresponding electrical conductance σ_{ij} using the Landauer formula.

If the energy of the highest occupied molecular orbital (HOMO) is E_H and the energy of the lowest unoccupied molecular orbital (LUMO) is E_L, then provided the source-drain voltage and the temperature are small compared with the HOMO-LUMO gap $\Delta = E_L - E_H$, the Landauer formula reduces to

$$\sigma_{ij} \approx G_0 T_{ij}(E_F) \qquad \text{(H.1)}$$

where E_F is the Fermi energy of the electrodes and G_0 is the quantum of conductance, given by $G_0 = \frac{2e^2}{h} \approx 77\mu S$, where e is the charge of an electron and h is Planck's constant.

If E_F lies in the vicinity of the middle of the HOMO-LUMO gap and the electrodes are weakly coupled to the electrodes, such that the level broadening due to the electrodes is small compared with Δ, then $T_{ij}(E)$ is proportional to $[g_{ij}(E)]^2$, where $g_{ij}(E)$ is the Green's function of the molecule, obtained by setting the coupling to the electrodes to zero.

Ie

$$T_{ij}(E) \approx A\tau_{ij}(E) \qquad \text{(H.2)}$$

where A is a constant, independent of i,j and

$$\tau_{ij}(E) = [g_{ij}(E)]^2 \,. \qquad \text{(H.3)}$$

$\tau_{ij}(E)$ will be referred to as a 'core transmission coefficient', because in the limit that the macrocycle is weakly coupled to the electrodes and for small values of E, the transmission coefficient $T_{ij}(E)$ of a macrocycle connected to external electrodes is proportional to $\tau_{ij}(E)$.

As described in chapter 2 of [textbook], $g_{ij}(E)$ is simply the amplitude of wave function at location i created by a source at location j and therefore reflects the interference pattern of a de Broglie wave of energy E created inside the molecule. If there are N possible injection and collection points, then for a given choice of E, $g_{ij}(E)$ can be regarded as the i,j th element of a NxN table (ie matrix) of numbers denoted $g(E)$.

For comparison with experiment, calculations of the connectivity dependence of transport properties are based typically on density functional theory or on tight-binding (ie Hückel) theory. If H is the mean-field Hamiltonian of the molecule, obtained by setting the coupling to the electrodes to zero, then the matrix $g(E)$ is given by

$$g(E) = (E - H)^{-1} \qquad \text{(H.4)}$$

where I is the unit matrix. Since the inverse of a matrix can be computed via a single line of eg a MATLAB code (ie $g = inv(E * I - H)$), the problem of computing the connectivity dependence of transport properties within the HOMO-LUMO gap, at low temperature and in

the presence of weakly coupled electrodes reduces to that of adopting a suitable Hamiltonian H.

For quantitative comparison with experiment, this can be obtained from density functional theory. However such calculations provide little insight into the underlying QI effects. To reveal the latter, it is useful to start from a tight-binding (ie Hückel) description of the molecule. When dealing with conjugate organic molecules with extended pi systems, this is achieved by assigning 'sites' to each π_z orbital. If the energy of an electron occupying site l is H_{ll} and the resonance integral (ie coupling) between sites l and m is H_{lm} are specified, then $g_{ij}(E)$, $T_{ij}(E)$ and hence the conductance σ_{ij} can be computed from equations (H.1) to (H.4). The presence of the unknown constant A in equation (H.2) means that absolute values of conductance cannot be calculated, but since A cancels in conductance ratios such as σ_{ij}/σ_{lm}, the above theory is ideal for understanding the relative change in conductance that occurs when the connectivity is changed from i,j to l,m.

For polyaromatic hydrocarbons, the simplest description is obtained by setting $H_{ll} = \epsilon_0$, where ϵ_0 is a constant, independent of the site index l. Also, if l,m are nearest neighbours, one chooses $H_{lm} = -\gamma$, where γ is a positive constant, and if l,m are not nearest neighbours, one chooses $H_{lm} = 0$. For such a simple model, if ϵ_0 is chosen to be the energy origin and γ is chosen to be

the energy scale, then provided E is measured relative to ϵ_0 in units of γ, one can choose $\epsilon = 0$ and $\gamma = 1$. With this choice, the Hamiltonian $H = -C$, where C is a simple a connectivity table, obtained by placing the number 1 at entries C_{lm} corresponding to nearest neighbours l,m and placing zeros elsewhere.

When utilising such a simple Hamiltonian, the resulting table

$$g(0) = -H^{-1} = C^{-1} \quad \text{(H.5)}$$

is called a 'magic number table' and denoted M. More generally, one defines

$$M = Bg(0), \quad \text{(H.6)}$$

where B is any constant, chosen for convenience. In what follows, we choose $B = 1$ and therefore

$$M = g(0) = C^{-1} \quad \text{(H.7)}$$

Clearly such a description only contains information the intra-molecular connectivity of π_z orbitals and does not contain information about the chemical nature of atoms within a molecule, other than assuming that such details are irrelevant. It is called 'magic number theory' and M is called a magic number table, because 'it is as if by magic' that such a simple description can

yield predictions about experimental measurements of electrical conductances of real molecules at room temperature.

As an example, figure S3 shows the connectivity table for the 6-ring macrocycle of figure 3a. For such a bipartite lattice, where odd-numbered sites connect to even-numbered sites only and vice versa, C is block off-diagonal, of the form

$$C = \begin{pmatrix} 0 & \overline{C}^t \\ \overline{C} & 0 \end{pmatrix} \quad \text{(H.8)}$$

(where $\overline{C}^t$ is the transpose of $\overline{C}$)and therefore only the sub-matrix $\overline{C}^t$is shown in figure S3. For comparison, the connectivity table for the corresponding linear chain of six rings shown is obtained by setting the coupling between the left-most and right-most sites to zero, because in a linear chain, sites 6 and 33 are not nearest neighbours. Ie for a linear chain, $C_{6,33} = C_{33,6} = 0$, which reflects the free-end boundary conditions of the chain.

From equation (6), $MC = 1$ and therefore M Is also block off-diagonal and of the form

$$M = \begin{pmatrix} 0 & \overline{M} \\ \overline{M}^t & 0 \end{pmatrix}, \quad \text{(H.9)}$$

where $$\overline{M} = \overline{C}^{-1} \quad \text{(H.10)}$$

For such a bipartite lattice, the middle of the HOMO-LUMO gap is located at $E = 0$ and therefore (see equations 1-3, 7) $\tau_{ij}(0) = (M_{ij})^2$ yields a theory of mid-gap transport.

This is discussed in appendix E, for a bipartite lattice,

$$g(E) \approx M + ED, \quad \text{(H.11)}$$

where D is a block-diagonal matrix of the form

$$D = \begin{pmatrix} \overline{D} & 0 \\ 0 & \overline{D}^t \end{pmatrix} \quad \text{(H.12)}$$

$$\overline{D} = \overline{M}\overline{M}^t \quad \text{(H.13)}$$

Hence if i is odd and j is even, or vice versa, corresponding to CQI connectivities,

$$g_{ij}(E) \approx M_{ij} \text{ plus a term of order } \left(\frac{E}{\Delta}\right)^2 \quad \text{(CQI)} \quad \text{(H.14)}$$

and if i,j are both odd or both even, corresponding to DQI connectivities,

$$g_{ij}(E) \approx -ED_{ij} \quad \text{(DQI)} \quad \text{(H.15)}$$

Equation (15) shows that near the middle of the HOMO-LUMO gap, for a bipartite lattice, the core transmission function for DQI connectivities is proportional to E^2 and of the form

$$\tau_{ij}(E) \approx E^2[D_{ij}]^2 \quad \text{(DQI)} \quad \text{(H.16)}$$

whereas for CQI connectivities is proportional to E^2 and of the form

$$\tau_{ij}(E) \approx [M_{ij}]^2 \quad \text{(CQI)} \quad \text{(H.17)}$$

References

(1) Lambert, C. J.; Liu, S. A Magic Ratio Rule for Beginners: A Chemist's Guide to Quantum Interference in Molecules. Chem. Eur. J. 2018, 24 (17), 4193–4201.

(2) Yoshizawa, K. An Orbital Rule for Electron Transport in Molecules. Acc. Chem. Res.

2012, 45 (9), 1612–1621.

(3) Lambert, C. J. *Quantum Transport in Nanostructures and Molecules*; IOP Publishing, 2021.

(4) Lambert, C. J.; Liu, S. A Magic Ratio Rule for Beginners: A Chemist's Guide to Quantum Interference in Molecules. *Chem. Eur. J.* **2018**, *24* (17), 4193–4201.

(5) Sangtarash, S.; Huang, C.; Sadeghi, H.; Sorohhov, G.; Hauser, J.; Wandlowski, T.; Hong, W.; Decurtins, S.; Liu, S. X.; Lambert, C. J. Searching the Hearts of Graphene-like Molecules for Simplicity, Sensitivity, and Logic. *J. Am. Chem. Soc.* **2015**, *137* (35), 11425–11431.

(6) Geng, Y.; Sangtarash, S.; Huang, C.; Sadeghi, H.; Fu, Y.; Hong, W.; Wandlowski, T.; Decurtins, S.; Lambert, C. J.; Liu, S.-X. Magic Ratios for Connectivity-Driven Electrical Conductance of Graphene-like Molecules. *J. Am. Chem. Soc.* **2015**, *137* (13), 4469–4476.

(7) Soler, J. M.; Artacho, E.; Gale, J. D.; García, A.; Junquera, J.; Ordejón, P.; Sánchez-Portal, D. The SIESTA Method for Ab Initio Order-N Materials Simulation. *J. Phys. Condens. Matter* **2002**, *14* (11), 2745–2779.

(8) Perdew, J. P.; Burke, K.; Ernzerhof, M. Generalized Gradient Approximation Made

Simple. *Phys. Rev. Lett.* **1996**, *77* (18), 3865.
